# Multiple Dynamical Mechanisms of Phase-2 Early Afterdepolarizations in a Human Ventricular Myocyte Model: *Involvement of Spontaneous SR Ca*^2+^ *Release*

**DOI:** 10.3389/fphys.2019.01545

**Published:** 2020-01-10

**Authors:** Yasutaka Kurata, Kunichika Tsumoto, Kenshi Hayashi, Ichiro Hisatome, Yuhichi Kuda, Mamoru Tanida

**Affiliations:** ^1^Department of Physiology II, Kanazawa Medical University, Uchinada, Japan; ^2^Department of Cardiovascular and Internal Medicine, Graduate School of Medical Sciences, Kanazawa University, Kanazawa, Japan; ^3^Department of Genetic Medicine and Regenerative Therapeutics, Graduate School of Medical Sciences, Tottori University, Yonago, Japan

**Keywords:** early afterdepolarization, spontaneous SR Ca^2+^ release, long QT syndrome, mathematical model, bifurcation analysis

## Abstract

Early afterdepolarization (EAD) is known to cause lethal ventricular arrhythmias in long QT syndrome (LQTS). In this study, dynamical mechanisms of EAD formation in human ventricular myocytes (HVMs) were investigated using the mathematical model developed by ten Tusscher and Panfilov (*Am J Physiol Heart Circ Physiol* 291, 2006). We explored how the rapid (I_Kr_) and slow (I_Ks_) components of delayed-rectifier K^+^ channel currents, L-type Ca^2+^ channel current (I_Ca__L_), Na^+^/Ca^2+^ exchanger current (I_NCX_), and intracellular Ca^2+^ handling via the sarcoplasmic reticulum (SR) contribute to initiation, termination and modulation of phase-2 EADs during pacing in relation to bifurcation phenomena in non-paced model cells. Parameter-dependent dynamical behaviors of the non-paced model cell were determined by calculating stabilities of equilibrium points (EPs) and limit cycles, and bifurcation points to construct bifurcation diagrams. Action potentials (APs) and EADs during pacing were reproduced by numerical simulations for constructing phase diagrams of the paced model cell dynamics. Results are summarized as follows: (1) A modified version of the ten Tusscher-Panfilov model with accelerated I_CaL_ inactivation could reproduce bradycardia-related EADs in LQTS type 2 and β-adrenergic stimulation-induced EADs in LQTS type 1. (2) Two types of EADs with different initiation mechanisms, I_CaL_ reactivation–dependent and spontaneous SR Ca^2+^ release–mediated EADs, were detected. (3) Termination of EADs (AP repolarization) during pacing depended on the slow activation of I_Ks_. (4) Spontaneous SR Ca^2+^ releases occurred at higher Ca^2+^ uptake rates, attributable to the instability of steady-state intracellular Ca^2+^ concentrations. Dynamical mechanisms of EAD formation and termination in the paced model cell are closely related to stability changes (bifurcations) in dynamical behaviors of the non-paced model cell, but they are model-dependent. Nevertheless, the modified ten Tusscher-Panfilov model would be useful for systematically investigating possible dynamical mechanisms of EAD-related arrhythmias in LQTS.

## Introduction

Early afterdepolarization (EAD) is well known to trigger lethal ventricular arrhythmias, called Torsades de Pointes (TdP), in patients with long QT syndrome (LQTS) (Weiss et al., 2010; Shimizu and Horie, 2011; Shimizu, 2013). For prevention and treatment of ventricular arrhythmias in LQTS patients, therefore, elucidating the mechanisms of initiation and termination of EADs and how to suppress EADs is of crucial importance. There are many experimental studies regarding the mechanisms of EAD formation in cardiomyocytes, suggesting major contribution of reactivation of the L-type Ca^2+^ channel current (I_CaL_) to the initiation of EADs during the action potential (AP) phase 2 (e.g., January et al., 1988; January and Riddle, 1989; Guo D. et al., 2007; Weiss et al., 2010; Xie et al., 2010; Milberg et al., 2012a; Shimizu, 2013). However, recent experimental studies suggested the major role in EAD formation of the spontaneous Ca^2+^ release from the sarcoplasmic reticulum (SR) (Volders et al., 2000; Choi et al., 2002; Zhao et al., 2012). In our recent theoretical study (Kurata et al., 2017) using two human ventricular myocyte (HVM) models developed by Kurata et al. (2005) and O’Hara et al. (2011), referred to as K05 and O11 models, respectively, we could find EAD formations resulting from the I_CaL_ reactivation, but not the spontaneous SR Ca^2+^ release-mediated EADs. With respect to the termination of EADs (AP repolarization), theoretical studies (Tran et al., 2009; Qu et al., 2013) using a guinea-pig ventricular myocyte model (Luo and Rudy, 1991) suggested the slowly activating delayed-rectifier K^+^ channel current (I_Ks_) as a key current to cause termination of EADs. However, our preceding study (Kurata et al., 2017) suggested that the mechanisms of EAD termination were model-dependent, not necessarily requiring I_Ks_. Thus, despite many experimental and theoretical studies, how individual membrane and intracellular components contribute to the initiation, termination and modulation of EADs remains controversial.

The aims of this study were (1) to determine whether the ten Tusscher and Panfilov model (ten Tusscher and Panfilov, 2006; referred to as the TP06 model) for HVMs, which has often been used for simulations of reentrant arrhythmias in the human ventricle (ten Tusscher et al., 2007; Adeniran et al., 2012; Zimik et al., 2015; Kazbanov et al., 2016), could reproduce EAD formation in LQTS (validation of the model cell for EAD reproducibility), and (2) to define the contributions of individual sarcolemmal and intracellular components to the initiation, termination, and modulation of phase-2 EADs in the TP06 model in comparison with those in other HVM models (evaluation of model dependence for EAD mechanisms). As in our preceding study (Kurata et al., 2017; Tsumoto et al., 2017), we examined parameter-dependent changes in stabilities of steady states and AP dynamics in the HVM model from the aspect of bifurcation phenomena, which are parameter-dependent qualitative changes in dynamical behaviors, in non-linear dynamical systems (Guckenheimer and Holmes, 1983; Parker and Chua, 1989; Kuznetsov, 2003). Conditions and dynamical mechanisms of EAD formation in the paced model cell were determined in relation to bifurcations of the non-paced model cell.

With respect to the dynamical mechanisms of EAD formation, we particularly focused on (1) whether and how contributions of each cellular component to occurrences of EADs and bifurcations in the TP06 model are different from those in the K05 and O11 models; (2) whether spontaneous SR Ca^2+^ release-mediated EAD initiation, which did not occur in the K05 or O11 model, can be reproduced by the TP06 model in connection with a bifurcation (destabilization) of intracellular Ca^2+^ dynamics; and (3) how slow I_Ks_ activation, as well as I_CaL_ inactivation and other slow factors, contributes to EAD termination. This study would further provide a theoretical background for experimental and simulation studies on mechanisms of EAD formation and EAD-triggered reentrant arrhythmias in the LQTS human ventricle, as well as for prevention and treatments of life-threatening arrhythmias, like TdPs, in LQTS.

## Materials and Methods

### Mathematical Modeling for HVMs

#### Base Mathematical Model

**FIGURE 1:**
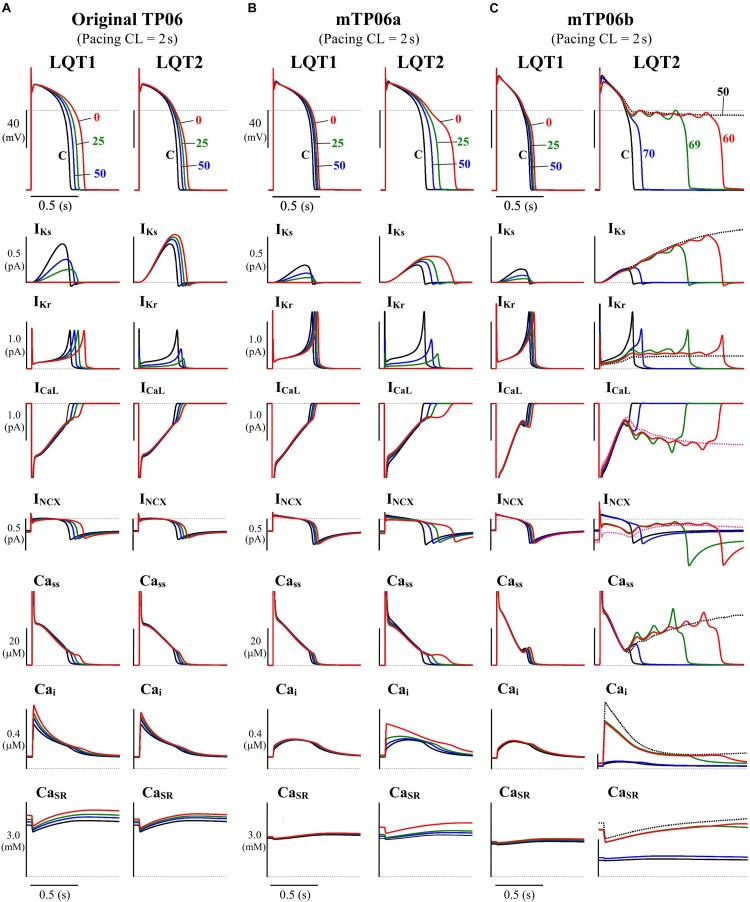
Simulated behaviors of APs (EADs), sarcolemmal ionic currents (I_Ks_, I_Kr_, I_CaL_, I_NCX_) and intracellular Ca^2+^ concentrations (Ca_ss_, Ca_i_, Ca_SR_) in the M cell versions of the original TP06 **(A)**, mTP06a **(B)**, and mTP06b **(C)** models. To mimic the pathological conditions of LQT1 and LQT2, g_Ks_ and g_Kr_ values, respectively, were decreased by 30–100%; individual APs are labeled by the numbers representing the residual g_Ks_ or g_Kr_ (%Control), with ionic currents and Ca^2+^ concentrations for each g_Ks_ and g_Kr_ value shown by the same colors. The horizontal dashed lines denote the 0 mV, zero current and zero concentration levels. Current amplitudes for the *y*-axis scale bars are given in pA/pF. The model cells were paced at 0.5 Hz, i.e., with the cycle length (CL) of 2 s for 60 min; AP waveforms, ion currents and Ca^2+^ concentrations after the last stimulus are shown as steady-state behaviors under each condition.

In this study, we tested the mid-myocardial (M) cell version of the TP06 model for HVMs (ten Tusscher and Panfilov, 2006), which could reproduce phase-2 EADs during inhibition of I_Ks_ and/or the rapidly activating delayed rectifier K^+^ channel current (I_Kr_) or enhancement of I_CaL_. The M cell version was chosen because it has smaller I_Kr_ and I_Ks_ and thus more vulnerable to EAD formation than the epicardial or endocardial version, as suggested experimentally as well (Antzelevitch et al., 1999), and a few modifications were made for the M cell version of the TP06 model. Figure 1 shows simulated behaviors of APs, sarcolemmal ionic currents and intracellular Ca^2+^ concentrations in the original and modified M cell versions of the TP06 model with various g_Ks_ and g_Kr_ values. Inconsistent with experiments for HVMs that observed only small prolongation of AP duration (APD) by I_Ks_ inhibition (Jost et al., 2005; O’Hara and Rudy, 2012), the original version of the TP06 model, which has relatively large I_Ks_, exhibited marked APD prolongation during I_Ks_ inhibition, and failed to reproduce greater APD prolongation and phase-2 EADs during I_Kr_ inhibition (see Figure 1A). In addition, the Ca^2+^ concentration in the SR (Ca_SR_) (3–4 mM during 1-Hz pacing) was higher than the experimentally observed values of 1–2 mM for rabbit ventricular myocytes (Shannon et al., 2003, 2004; Guo T. et al., 2007). Therefore, the modified version, referred to as the “mTP06a” model, underwent the following modifications: (1) 60% reduction of the maximum I_Ks_ conductance (g_Ks_) with 50% increment of the maximum I_Kr_ conductance (g_Kr_) to reproduce the I_Kr_/I_Ks_ inhibition experiments, and (2) 40% reduction in the SR Ca^2+^ uptake rate (P_up_) to reduce the Ca^2+^ concentration in the SR during pacing under control conditions. These modifications yielded the experimentally observed small APD prolongation by I_Ks_ inhibition and smaller Ca_SR_ of 1.3–2.6 mM during pacing at 0.5–1 Hz, but not EAD formation (Figure 1B). Therefore, we have developed another version of the modified TP06 model referred to as the “mTP06b” model with halved time constant of I_CaL_ inactivation (τ_fL_) and doubled maximum I_CaL_ conductance (g_CaL_) on the basis of a previous theoretical study by Vandersickel et al. (2014) that required acceleration of the voltage-dependent inactivation of I_CaL_ for reproducing EADs in the TP06 model. As shown in Figure 1C, the mTP06b model could reproduce the experimentally observed responses of HVMs to reductions of I_Kr_ or I_Ks_, with EADs generated during I_Kr_ reductions. Maximum conductance of the ionic channels, densities of transporters, and SR Ca^2+^ uptake/release rates for the modified versions, as well as for the original version, are given in Supplementary Table S1.

The TP06 model for the normal activity of single HVMs is described as a non-linear dynamical system of 19 first-order ordinary differential equations. The membrane current system includes the Na^+^ channel current (I_Na_), I_CaL_, I_K__r_, I_Ks_, 4-aminopyridine-sensitive transient outward current (I_to_), inward-rectifier K^+^ channel current (I_K__1_), background K^+^ (I_pK_), Na^+^ (I_b__Na_) and Ca^2+^ (I_b__C__a_) currents, Na^+^-K^+^ pump current (I_NaK_), Na^+^/Ca^2+^ exchanger current (I_N__CX_), and Ca^2+^ pump current (I_pCa_). Time-dependent changes in the membrane potential (V_m_) are described by the equation,

dV/mdt=I-stim(I+NaI+CaLI+KrI+KsI+to

(1) I+K1I+pKI+bNaI+bCaI+NaKI+NCXI)pCa

where I_stim_ represents the stimulus current (in pA/pF).

The basic model systems include material balance expressions to define the temporal variations in concentrations of myoplasmic K^+^ (K_i_), Na^+^ (Na_i_) and Ca^2+^ (Ca_i_), and subspace Ca^2+^ (Ca_ss_), while external concentrations of K^+^, Na^+^ and Ca^2+^ were fixed at 5.4, 140, and 2.0 mM, respectively. For bifurcation analyses, K_i_ was fixed at 140 mM for the removal of *degeneracy* (Krogh-Madsen et al., 2005; Kurata et al., 2008); effects of parameter-dependent changes in K_i_ (∼5 mM) on EAD formation and bifurcation phenomena in the model cell were much smaller than those of the same amount of changes in Na_i_. Na_i_ was unfixed unless otherwise stated, but fixed at 6 mM in some cases (e.g., for the slow-fast decomposition analysis and for voltage-clamped cells, as described later); changes in Na_i_ during AP phase 2 and EAD formation in paced model cells were slow and relatively small.

Details on expressions, standard parameter values, and dynamics of the TP06 model are provided in the original article (ten Tusscher and Panfilov, 2006), and the original TP06 model has been implemented in a cellML-based open resource for public access^1^. In addition, the original TP06, mTP06a, and mTP06b models have been implemented in PhysioDesigner as XML-based Physiological Hierarchy Markup Language (PHML)^2^ models. These models can be referred from PHML database (ID938 to 940)^3^, and simulations of their temporal behaviors can be performed using the software, Flint^4^.

#### Modeling LQTS Cardiomyocytes With Simulated EADs

Mutations of I_K__s_ and I_K__r_ channels (Roden et al., 1996; Chouabe et al., 1997; Anderson et al., 2006; Wiener et al., 2008; Kondo et al., 2016), as well as their pharmacological inhibitions (e.g., Carmeliet, 1992; Volders et al., 2003; Jost et al., 2005), are known to cause a wide range of channel conductance changes, leading to congenital or acquired LQTS type 1 (LQT1) and type 2 (LQT2), respectively. We developed LQT1- and LQT2-type model cells by continuously reducing g_Ks_ and g_Kr_, respectively, from unity to zero. As illustrated in Figure 1, the TP06b model, but not the original version (with larger I_Ks_) or the mTP06a model, reproduced phase-2 EADs (and AP repolarization failure) when g_Kr_ became smaller as in LQT2 cardiomyocytes. In contrast, g_K__s_-reduced LQT1 model cells did not exhibit EADs but showed only slight prolongation of APDs under the basal condition, consistent with the recent experimental results from HVMs (Jost et al., 2005; O’Hara and Rudy, 2012).

#### Simulating Conditions of β-Adrenergic Stimulation

To simulate the condition of β-adrenergic stimulation (β-AS) as a major trigger of EADs and TdP in LQT1 patients, we modified the maximum conductance of ion channels and density of transporters based on previous reports (Zeng and Rudy, 1995; Volders et al., 2003; Kuzumoto et al., 2008), as described in our preceding article (Kurata et al., 2017). g_CaL_ and g_Ks_ were increased up to 250% and 200%, respectively, according to previous reports for their changes during β-AS (Veldkamp et al., 2001; Saucerman et al., 2003; Himeno et al., 2008; Maltsev and Lakatta, 2010; Briston et al., 2014). Modifications of parameters for simulating β-AS are listed in Supplementary Table S2.

### Numerical Methods for Dynamic Simulations

#### Basic Methods

Dynamic behaviors of the model cells were determined by numerically solving a set of non-linear ordinary differential equations including Eqn. 1. AP responses were elicited by 1-ms current stimuli of 60 pA/pF. When phase-2 EADs occurred at higher frequencies of the pacing, a complete AP repolarization was preceded by the next stimulus. Thus, the pacing cycle length (CL) was usually set to longer values of 2–5 s, except for analyses of the rate dependence. Numerical integration was performed by using MATLAB (The MathWorks, Inc., Natick, MA, United States) ODE solvers, *ode15s* and *ode45*, with the maximum relative error tolerance for the integration methods of 1 × 10^–8^.

Initial values of the state variables for computation at a parameter set were their steady-state values at a resting V_m_ (see Supplementary Table S3 for the control conditions), which were perturbed by the current stimulus; the last values of the state variables in computation were used as initial conditions for the next computation at a new parameter set. The minimum V_m_ during AP phase 4 (V_min_) and the maximum V_m_ during early phase 2 before emergence of an EAD (V_max_), as well as APD at 90% repolarization (APD_90_), were determined for individual APs or AP sets. Steady-state APs for the first parameter set were obtained by numerical integration for 30 min; subsequent numerical integration with each parameter set was continued until the differences in V_min_, V_max_ and APD_90_ between the newly calculated AP and the preceding one became <1 × 10^–3^ of their preceding values.

#### Detection of EADs

EADs were detected as transient V_m_ oscillations which emerged during late AP phase 2 (200 ms or later from the AP peak) and eventually led to AP repolarization to a resting V_m_. All the local minimum (EAD_min_) and maximum (EAD_max_) of V_m_ oscillations during EAD formation, as well as a set of V_min_, V_max_ and APD_90_, were determined for one AP cycle. When APs with EADs were irregular (arrhythmic), all the potential extrema (V_min_, V_max_, EAD_min_, and EAD_max_) and APD_90_ values were sampled for APs evoked by the last 10 stimuli.

### Stability and Bifurcation Analyses for HVMs

We performed bifurcation analysis to explore how dynamical properties of the HVM model cell systems alter with changes in parameters. Detailed procedures for bifurcation analyses, i.e., locating equilibrium points (EPs) and limit cycles (LCs), detecting bifurcation points by determination of their stabilities, were provided in our previous articles (Kurata et al., 2008, 2012, 2013, 2017; Tsumoto et al., 2017), as well as in textbooks (Guckenheimer and Holmes, 1983; Parker and Chua, 1989; Kuznetsov, 2003). In the present study, one- and two-parameter bifurcation diagrams for the non-paced cell model, as well as phase diagrams for the paced model cell, were constructed as functions of parameters, including (1) g_Ks_, g_Kr_, and g_CaL_, (2) scaling factor for I_NCX_, (3) P_up_, and (4) pacing CL. The maximum conductance of the ionic channel currents and P_up_ were expressed as normalized values, i.e., ratios to the control values. Mechanisms of the initiation and termination of EADs were further examined by the *slow-fast decomposition analysis*, in which stability and bifurcations of a fast subsystem are determined as functions of a slow variable, i.e., the gating variable *xs* for I_Ks_ activation or Ca_SR_ (Tran et al., 2009; Qu et al., 2013; Xie et al., 2014). Basic concepts of bifurcation analysis, types of bifurcations, and methods for constructions of bifurcation/phase diagrams and slow-fast decomposition analysis are briefly described in Supplementary Materials.

## Results

### Validation and Characterization of the mTP06 Models for LQTS HVMs

We first determined whether the mTP06a/b models can mimic the electrophysiological properties of I_Kr_-reduced LQTS type 2 (LQT2) and I_Ks_-reduced LQTS type 1 (LQT1) HVMs, in which EADs occur mainly at lower heart rates (bradycardia), and under β-AS, e.g., during exercise (tachycardia), respectively.

#### Decreases in I_Kr_ and/or I_Ks_ Accelerated EAD Formation in the mTP06 Model

**FIGURE 2:**
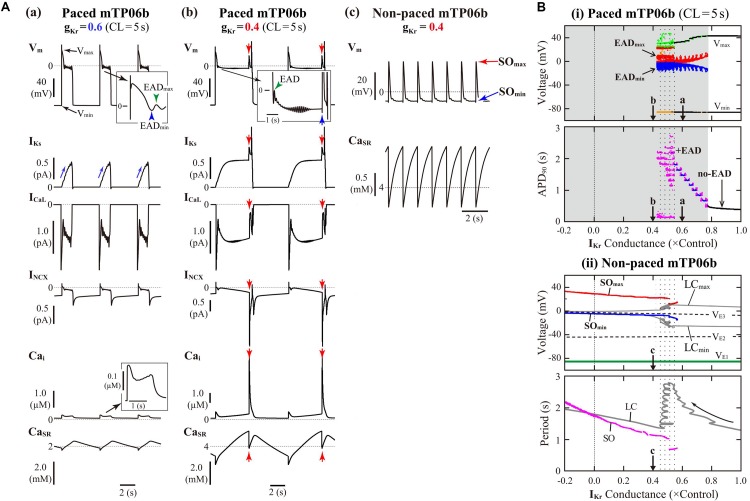
I_Kr_-dependent EAD generation and bifurcations in the mTP06b model. **(A)** Simulated dynamics of V_m_, I_Ks_, I_CaL_, I_NCX_, Ca_i_, and Ca_SR_ (from *top* to *bottom*) in the g_Kr_-reduced model cells paced at 0.2 Hz, illustrating two types of EADs. Temporal behaviors of the variables were computed for 30 min; the behaviors elicited by additional 3-4 stimuli are shown. APs with EADs were terminated (V_m_ repolarized) by gradual increases in I_K__s_ **(a)** or the next stimulus as indicated by the blue arrow **(b)**. Spontaneous SR Ca^2+^ releases and resulting increases of inward I_NCX_ to provoke EADs occurred at g_K__r_ = 0.4, as indicated by the red arrows **(b)**. With the smaller g_K__r_, sustained V_m_ oscillations driven by spontaneous SR Ca^2+^ releases were observed after cessation of pacing, i.e., in the non-paced model cell **(c)**. **(B)** Potential extrema of APs and EADs, and the AP duration (APD) measured at 90% repolarization (APD_90_) are plotted as functions of normalized g_Kr_ for the paced model cell **(i)**. One-parameter bifurcation diagrams depicting steady-state V_m_ at equilibrium points (EPs), the extrema of limit cycles (LCs) and spontaneous oscillations (SOs), and the periods of LCs and SOs plotted against g_Kr_ are also shown for the non-paced model cell **(ii)**. In the panel **(i)**, AP dynamics during 0.2-Hz pacing were computed for 1 min at each g_Kr_ value, which was reduced from 1.0 to −0.2 at an interval of 0.001. The minimum V_m_ during AP phase 4 (V_min_) and the maximum V_m_ during AP phase 2 before EAD formation (V_max_) are indicated by black dots for rhythmic APs, and by orange (V_min_) and light green (V_max_) dots for arrhythmic APs. When EADs appeared, their local potential minimum (EAD_min_) and maximum (EAD_max_) were plotted by blue and red dots, respectively. In the panel for APD_90_, the black, blue, and magenta dots represent APD_90_ values for regular APs without EAD, regular APs with EADs, and arrhythmic APs with EADs, respectively (no-EAD: APs without EAD, + EAD: APs with EADs). The points labeled as “**a**” and “**b**” indicate g_Kr_ values for which AP behaviors are shown in Panel **(A)**. In the panel **(ii)**, the steady-state branches as loci of V_m_ at EPs (V_E__1__–__3_), periodic branches as the potential minimum (LC_min_) and maximum (LC_max_) of LCs, and potential extrema of SOs (SO_min_, SO_max_), as well as the periods of LCs and SOs, are plotted for the non-paced model cell. The steady-state branch V_E__1_ is stable (green solid lines), while V_E__2_ and V_E__3_ unstable (black dashed lines). The periodic branches represented by gray solid lines are always unstable. The point labeled as “**c**” indicates the g_Kr_ values for which SOs are shown in **A-c**.

The mTP06b model, but not the original TP06 or mTP06a model, exhibited an AP with EADs when I_Kr_ was inhibited during 0.5-Hz pacing (Figure 1). Similarly, when g_Kr_ was reduced by 40% during 0.2-Hz pacing, we could observe the AP with EADs in the g_Kr_-reduced mTP06b model, as shown in Figure 2A-a. This simulated AP with EADs was accompanied by oscillatory reactivation of I_CaL_, and was terminated (i.e., V_m_ went back to the resting V_m_) as I_Ks_ increased (blue arrows for I_Ks_ in Figure 2A-a). Further reducing g_Kr_ by 60% caused another type of EADs with I_Ks_ saturated before applying the second stimulus (Figure 2A-b); just before applying this second stimulus, the transient depolarization in plateau phase originating from the spontaneous SR Ca^2+^ release and resulting activation of inward I_NCX_ (red arrows in Figure 2A-b). In this case, repolarization did not occur without the next stimulus, i.e., repolarization failure occurred in the non-paced model cell after the cessation of pacing (Figure 2A-c).

Figure 2B-i shows switching of AP dynamics when g_Kr_ was gradually reduced during 0.2-Hz pacing. In this figure for the paced system, V_m_ extrema of APs (V_min_/V_max_) and EADs (EAD_min_/EAD_max_), were plotted against g_Kr_. EADs emerged at g_Kr_ = 0.772, i.e., with 22.8% block of I_Kr_ (Figure 2B-i, *top*); the g_Kr_ reduction led to increases in the number of EADs, resulting in the discrete increase of APD_90_ values (see Figure 2B-i, *bottom*). The AP repolarization dynamics in the paced cell model relates to the dynamical behavior of the non-paced cell model because there is no stimulation during the AP repolarization. Therefore, we investigated the dynamical behavior of the non-paced cell model using bifurcation analysis. Figure 2B-ii shows one-parameter bifurcation diagrams as functions of g_Kr_, constructed for the non-paced mTP06b model (see also Supplementary Figure S1A showing those for the mTP06a model for comparison). In the non-paced mTP06b and mTP06a model, there existed three EPs as the steady states. The EP in the upper steady-state branch (V_E__3_ in Figure 2B-ii and Supplementary Figure S1A-ii) was always unstable at positive g_Kr_ values, while stable at negative g_Kr_ values in the mTP06a model. When g_Kr_ markedly reduced to a large negative value (out of range in Figure 2B-ii), the unstable EP (V_E__3_) underwent the supercritical Hopf bifurcation (HB), which changed it to a stable EP and led to a generation of LC oscillation. The LCs spawned from the HB point were always unstable in the positive g_Kr_ range (see gray lines in Figure 2B-ii and Supplementary Figure S1A-ii). On the one hand, we found small-amplitude spontaneous V_m_ oscillations (SOs) that occurred at depolarized V_m_ (red and blue lines in Figure 2B-ii, *top*) in the vicinity of the unstable LCs, as exemplified in Figure 2A-c. Just before the disappearance of SOs with increasing g_Kr_, the period of unstable LC markedly prolonged (see the gray zigzag trace in Figure 2B-ii, *bottom*). This marked prolongation of LC periods and the emergence of SOs correlated with very long APD (long-lasting EADs) and irregularity of the repolarization time in the paced cell model (compare the dotted ranges in Figures 2B-i,ii).

In contrast, V_E__3_ in the I_Ks_-eliminated (g_Ks_ = 0) non-paced model cells was stabilized via an occurrence of the subcritical HB when g_Kr_ was reduced (solid green lines to the left of the label “H” in Supplementary Figure S1B-ii). Thus, I_K__s_ inhibition caused drastic shift of HB points toward higher g_Kr_ values. Unstable LCs emerged via the subcritical HB, not changing their stability in the g_Kr_ range tested. In the I_Ks_-eliminated paced mTP06a/b models (Supplementary Figure S1B-i), decreasing g_Kr_ did not yield EADs, but abruptly changed APs without EADs to local responses in the depolarized V_m_ range during pacing, i.e., arrest at stable EPs (V_E__3_) without pacing.

**FIGURE 3:**
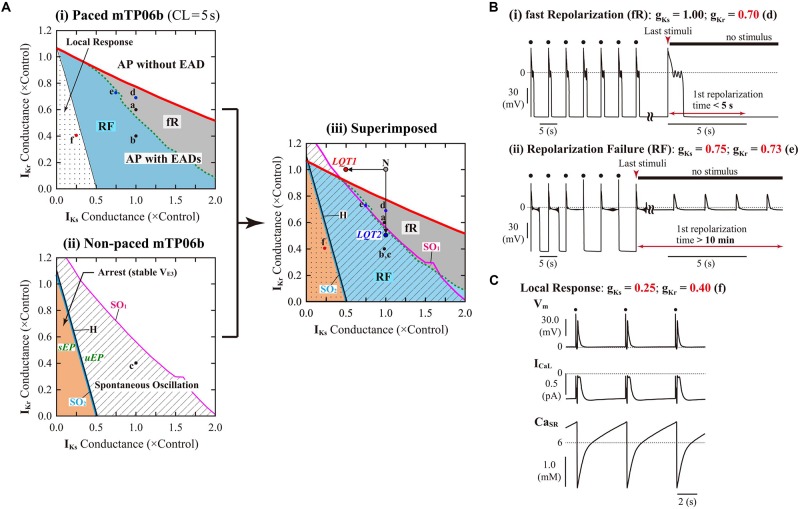
I_Kr_/I_Ks_-dependent EAD formation, dynamics and bifurcations of the mTP06b model. **(A)** A phase diagram indicating the region of EAD formation (and local responses) in the paced model cell **(i)** and two-parameter bifurcation diagrams for the non-paced model cell **(ii)** on the g_Ks_–g_Kr_ parameter plane. In the diagram for the paced model cell **(i)**, the thick red solid, black dashed and thin black solid lines, respectively, indicate parameter sets of critical points at which short-term EADs (APD_90_ < 5 s, as in Figure 2A-a and **[B-(i)]**, long-term or sustained EADs (APD_90_ > 5 s, as in Figure 2A-b and **[B-(ii)]**, and a local response **(C)** emerged; parameter regions in which short-term EADs, long-term or sustained EADs, and local responses occur are shown as the light-gray region labeled as “fR” (fast repolarization), blue region labeled as “RF” (repolarization failure), and dotted region, respectively. In the two-parameter bifurcation diagram for the non-paced model cell **(ii)**, H, SO_1_, and SO_2_ indicate parameter sets of HB points, critical points at which SOs emerged, and critical points at which SOs switched into quiescence, respectively. The parameter regions in which SOs and convergence to the steady state (V_E__3_), i.e., arrest, can be observed are indicated as the shaded and orange regions, respectively. The labels “*sEP*” and “*uEP*” indicate the areas of stable and unstable EPs, respectively, divided by the HB curve. The panel **(iii)** is the diagram for which the phase diagram **(i)** is superimposed upon the two-parameter bifurcation diagram **(ii)**. The points labeled as “N”, “*LQT1*” and “*LQT2*” denote the normal, LQT1, and LQT2 conditions, respectively. The points “**a**”–“**f**” indicate parameter sets for which AP behaviors are shown in Figures 2A-a–c, and **[B (d,e)] [C (f)]**. **(B)** Representative behaviors of APs with EADs during 0.2-Hz pacing at the points labeled as “**d**” and “**e**” in **(A)**, which are classified into the fast repolarization type **(d)** and repolarization failure type **(e)** behaviors, respectively. **(C)** An example of the local response during 0.2-Hz pacing at the point “**f**” in **(A)**.

To evaluate the dependencies of EAD formation on g_Kr_ and g_Ks_, we performed AP simulations using the mTP06b model with various sets of g_Kr_ and g_Ks_. Figure 3A-i shows a phase diagram of AP behaviors for changes in g_Ks_ and g_Kr_ values with 0.2-Hz pacing. By characterizing AP behaviors observed in the paced mTP06b model, the g_K__s_–g_K__r_ parameter plane was divided into three regions: (1) AP without EAD, (2) AP with EADs (colored regions; see examples of Figure 3B for the points “d” and “e” in Figure 3A-i), and (3) local response (dotted region; see an example of Figure 3C for the point “f” in Figure 3A-i). We further separated the region of the AP with EADs into two regions based on characteristics of the repolarization time in an AP with EADs: During 0.2-Hz pacing, further decreases in g_Kr_ (and/or g_Ks_) in the EAD region altered an AP with shorter APD_90_ of ≤5 s that repolarizes before the next stimulus to an AP with longer APD_90_ of >5 s that is repolarized by the next stimulus, as shown in Figure 2A; then, the APs with EADs were defined as “fast repolarization (fR)” type for the former and “repolarization failure (RF)” type for the latter, which are exemplified in Figures 2A-a,b, respectively. The fR and RF types were distinguished by AP behaviors after an extra stimulus following the last test stimulus to cause AP repolarization, as illustrated in Figure 3B: The fR-type AP repolarized to resting V_m_ within 5 s (Figure 3B-i), while the RF-type one did not (Figure 3B-ii); in this case, APD_90_ values of the RF-type AP were almost always more than 10 min.

Decreases in g_Kr_ and/or g_Ks_ required for EAD formation were much smaller in the mTP06b model than in the mTP06a model (compare Figure 3A-i and Supplementary Figure S2). The borderline of EAD initiation (the red solid line in Figure 3A-i) shifted in a g_Ks_-dependent manner, with the g_Kr_ region of EADs broadening as g_Ks_ increased. Furthermore, two-parameter bifurcation analysis for the non-paced cell model (Figure 3A-ii) determined three areas with different behaviors: (1) quiescence at a stable EP (resting state; V_E__1_) with no stable EP or LC at depolarized V_m_, (2) co-existence of quiescence at a stable EP (resting state) and a stable LC or SO at depolarized V_m_ (shaded area labeled as “Spontaneous Oscillation”), and (3) co-existence of two stable EPs at V_E__1_ and depolarized V_m_ (V_E__3_), i.e., the arrest at depolarized V_m_ (colored area labeled as “Arrest (stable V_E__3_)”).

To clarify relationships between AP responses observed in the paced cell model and bifurcations occurred in the non-paced cell model, we superimposed the phase diagram on the two-parameter bifurcation diagram (Figure 3A-iii). Most of the SO region in which SOs can be observed in the non-paced cell model was included in the RF region, suggesting the relation of spontaneous SR Ca^2+^ release-mediated sustained EADs to SOs (Figure 3B-ii). The borderline between local response and AP with EADs corresponded to the HB set in the non-paced cell model, indicating that V_m_ in the paced cell model converges to the stable EP (V_E__3_) in the area of local response.

#### Slow and Rapid Pacing Facilitated EAD Formation in the mTP06b Model

**FIGURE 4:**
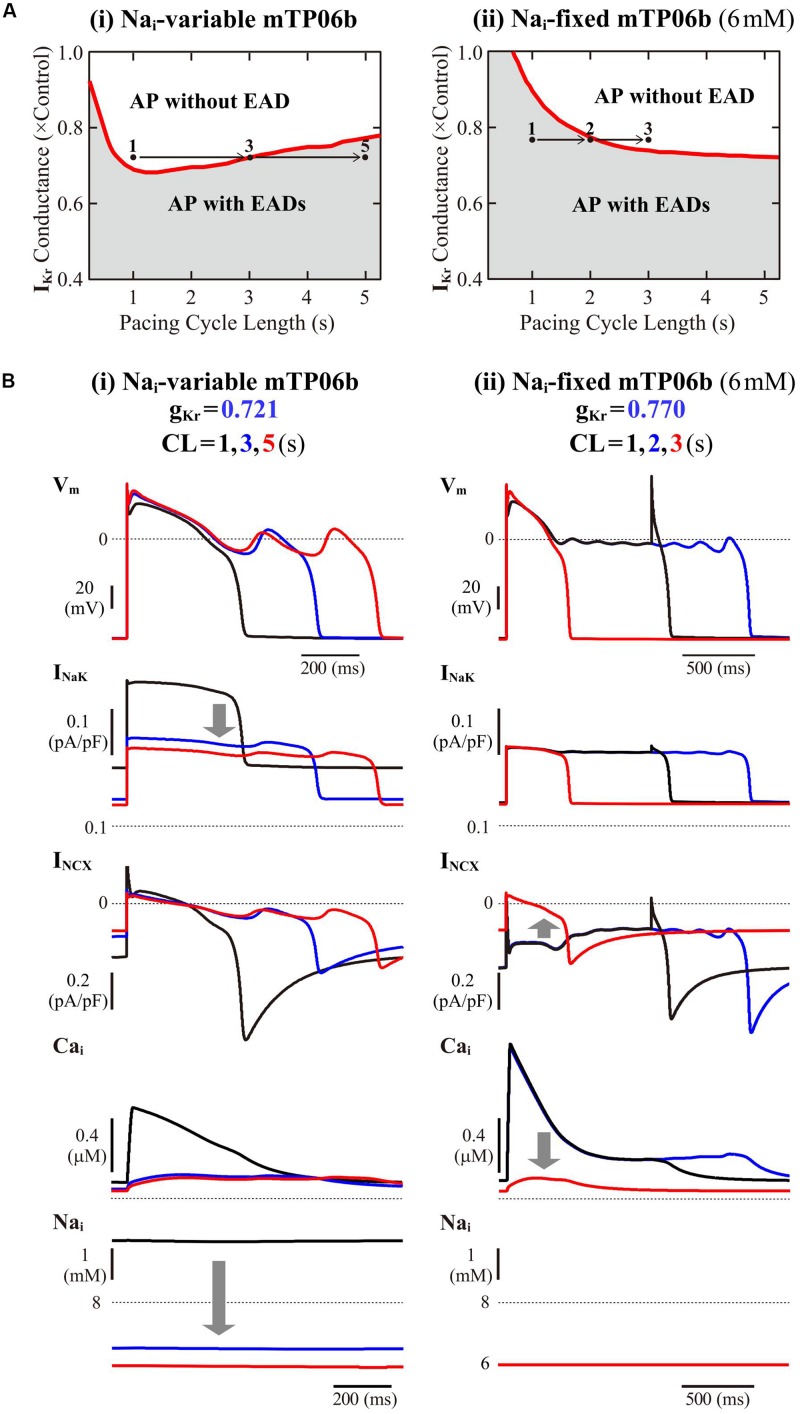
Rate dependence of EAD generation in the mTP06b model. **(A)** Two-parameter phase diagrams for the pacing cycle length (CL) and g_Kr_ depicted for the Na_i_-variable **(i)** and Na_i_-fixed **(ii)** model cells paced with various CLs of 0.75-5.25 s at 0.01-0.2 s intervals. The red solid lines and gray regions represent the parameter sets of critical points for occurrences of EADs and parameter region in which APs with EADs can be observed, respectively. **(B)** Simulated dynamics of the Na_i_-variable **(i)** and Na_i_-fixed **(ii)** g_Kr_-reduced model cell paced at various frequencies (with CLs of 1, 3, and 5 s or 1, 2, and 3 s). Temporal behaviors of the model cell were computed for 30 min at each pacing rate; V_m_, I_NaK_, I_NCX_, and Na_i_ for the last 1-2 s are shown as steady-state dynamics. The arrows indicate the directions of changes induced by increases in CL.

To further validate the mTP06b model as a LQT2 model, we next determined whether EAD formation in the g_Kr_-reduced mTP06b model is facilitated at lower pacing rates (in bradycardia). Rate effects on EAD formation are shown in the diagrams depicting the g_Kr_ regions of EADs as functions of the pacing cycle length (Figure 4A). EAD formation in the g_Kr_-reduced system was promoted at lower pacing rates in the Na_i_-variable system, while prevented in the Na_i_-fixed system. As in the K05 and O11 models (Kurata et al., 2017), the facilitation of EAD formation at lower pacing rates in the Na_i_-variable mTP06b model was accompanied by the decrease in Na_i_, which resulted in the decrease of outward I_NaK_ leading to delays in AP repolarization and EAD formation (Figure 4B-i). In the Na_i_-fixed mTP06b model, the inhibition of EAD formation at lower pacing rates accompanied marked outward shift of I_NCX_ resulting from diminished Ca_i_ transients (Figure 4B-ii). In Supplementary Figure S3, two-parameter bifurcation diagrams on the g_Ks_–g_Kr_ parameter plane are also shown for the Na_i_-variable and Na_i_-fixed mTP06b model cells paced at 0.2 and 1 Hz. In the Na_i_-variable system (Supplementary Figure S3A), slower pacing promoted EAD formation during decreases of g_Kr_ and/or g_Ks_ and broadened the parameter region of EADs; in the Na_i_-fixed system (Supplementary Figure S3B), however, the rate-dependent changes in the onset and region of EADs were opposite to those in the Na_i_-variable system (compare the gray and blue areas in each panel of Supplementary Figure S3).

The rate dependence shown in Figure 4 and Supplementary Figure S3 was determined by quasi steady-state dynamics for each parameter set. In LQT2 patients, however, EADs and TdP may often be induced by abrupt pause or transient slowing of heartbeats (bradycardia) at rest or during sleep. Thus, we also determined how EADs emerge after sudden reductions of pacing rates (Supplementary Figure S4). When a pacing CL was increased from 1 s to 3, 4, and 5 s in the I_Kr_-reduced mTP06b model (g_Kr_ = 0.721), EADs were first induced by 172nd, 74th, and 51st stimulus, respectively, after the reductions in pacing rates; pause-induced EAD or early onset of EADs after the increment in pacing CL was not observed, but long bradycardiac periods of more than 4 min were needed for EAD formation in this model cell.

In the mTP06b model, rapid pacing (CL < 1 s) also facilitated EAD formation via increases in Ca_i_ and Ca_SR_, and resulting increases of inward I_NCX_ (data not shown). EADs are known to be inhibited at higher pacing rates by accumulation of slowly deactivating I_Ks_ as well as reductions in I_CaL_ due to slow recovery. Cumulative I_Ks_ increments and I_CaL_ reductions were certainly observed at the higher pacing rates in the mTP06b model as well; however, the enhanced inward I_NCX_ appeared to cause APD prolongation and EAD formation in this model cell.

#### Spontaneous SR Ca^2+^ Release-Medicated EADs Occurred During β-AS

**FIGURE 5:**
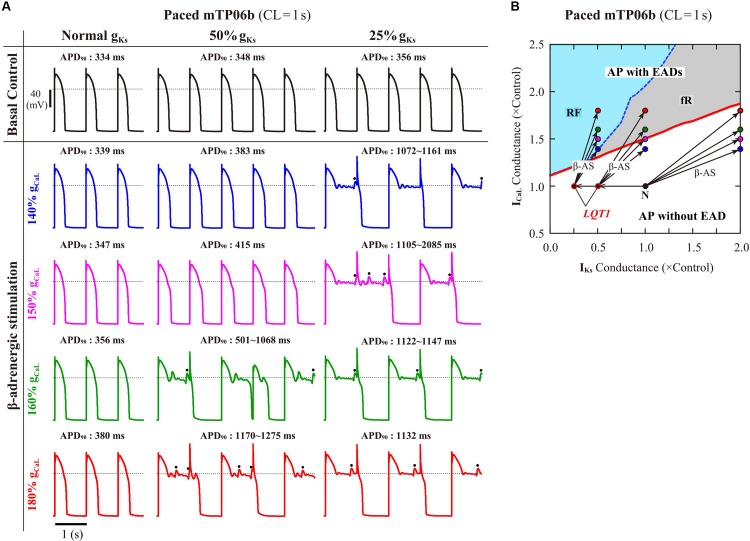
EAD generations during β-AS in the normal and LQT1 versions of the mTP06b model. **(A)** Simulated APs of the model cells under the basal condition (*top*) and conditions of β-AS as indicated by the points and arrows in **(B)**. Model cells were paced at 1 Hz for 30 min. The dots denote EADs induced by spontaneous SR Ca^2+^ releases. **(B)** A phase diagram on the g_Ks_–g_CaL_ parameter plane depicting displacements of critical points at which EADs emerged during 1-Hz pacing. The critical points were determined during g_CaL_ increases at an interval of 0.002 for individual g_Ks_ values increased at intervals of 0.02–0.1. For simulating the conditions of β-AS, the parameters other than g_CaL_ and g_Ks_ were modified as stated in the section “Materials and Methods” (see Supplementary Table S2). The LQT1 model cell was assumed to have reduced g_Ks_ of 50% or 25% of the control value. The points of the control (basal) conditions for cardiomyocytes with the normal and reduced g_Ks_ are labeled as “N” and “*LQT1*”, respectively. The arrows indicate the parameter shifts from the basal condition to the conditions of β-AS with g_Ks_ doubled and g_CaL_ increased to 140, 150, 160 and 180% of the control value.

To validate the mTP06b model as a LQT1 model, i.e., to determine whether the I_Ks_-reduced model cell can exhibit EADs under the conditions of β-AS, we examined susceptibilities to EAD generation during β-AS of the normal and LQT1 versions of the mTP06b model. For the LQT1 model cell, g_Ks_ was reduced by 50% and 75%, following the reports for the KCNQ1 mutations M437V and A590W, respectively (see Sogo et al., 2016). Figure 5A shows simulated APs of the normal and LQT1 versions of the mTP06b model under the basal condition and conditions of β-AS with g_CaL_ increased to 140, 150, 160, and 180% of the control value. The LQT1 model cells exhibited longer APDs under the basal condition (APD_90_ of 334 ms with the normal g_Ks_ vs. 348 ms with 50% g_Ks_ and 356 ms with 25% g_Ks_) and EADs under β-AS with g_CaL_ increased by 60% or more for 50% g_Ks_ and 40% or more for 25% g_Ks_, whereas the normal cell did not exhibit EAD. By constructing a two-parameter bifurcation diagram on the g_Ks_–g_CaL_ plane for the Na_i_-variable model cell paced at 1 Hz (Figure 5B), we could explain their EAD formation under the conditions of β-AS. As in the K05 model (Kurata et al., 2017), EAD formation during g_CaL_ increases under β-AS could be inhibited by concomitant g_Ks_ increases more effectively in the normal mTP06b model than in the LQT1 models: The LQT1 model cells entered the area of EAD formation with smaller increases in g_CaL_ (50.8% or more for the 50% g_Ks_ reduction and 31.0% or more for the 75% g_Ks_ reduction), while the normal cell with more than 87.7% increases in g_CaL_. Thus, the mTP06b model could recapitulate EAD formation via enhancement of I_CaL_ during β-AS in the LQT1 cardiomyocyte. Under β-AS with higher g_CaL_ and P_up_, spontaneous SR Ca^2+^ releases as evidenced by abrupt falls in Ca_SR_ without I_CaL_ reactivation often occurred, leading to Ca_i_ elevations (see Supplementary Figure S5), increments of inward I_NCX_, and resultant EADs (or spontaneous V_m_ oscillations), as indicated by the dots in Figure 5A.

#### Influences of SR Ca^2+^ Cycling, I_NCX_ and I_CaL_ on EAD Formation

**FIGURE 6:**
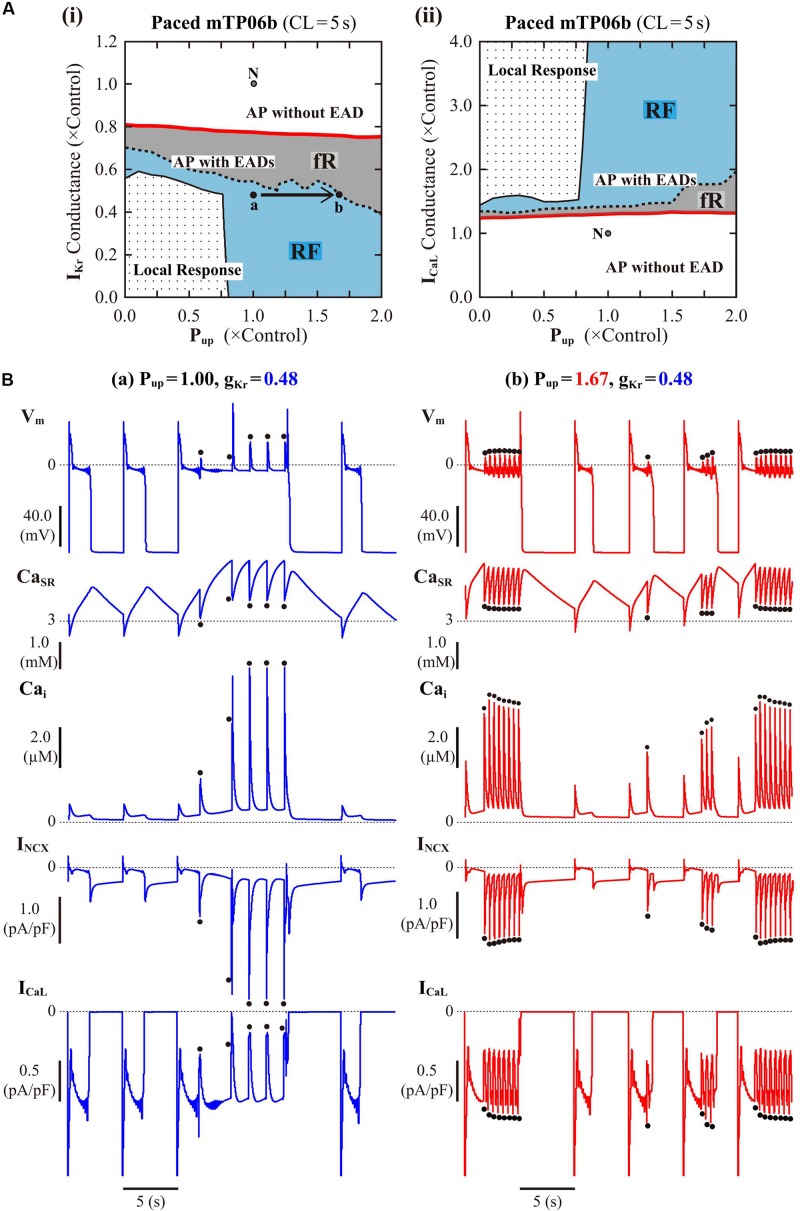
Influences of SR Ca^2+^ handling on EAD generation in the mTP06b model. **(A)** Phase diagrams on the P_up_–g_Kr_ **(i)** and P_up_–g_CaL_ **(ii)** parameter planes, depicting displacements of critical points for the occurrence of short-term EADs (red solid lines) and long-term or sustained EADs (dashed lines) as well as the emergence of local responses (black solid lines). By the parameter sets of these critical points, the parameter planes are divided into the areas of APs with short-term EADs (fR), APs with long-term or sustained EADs (RF) and local response, as described for Figure 3A. The points “**a**” and “**b**” in the panel **(i)** denote the parameter sets for simulations of the model cell dynamics shown in **B-a,b**, respectively. **(B)** Simulated dynamics of the model cells with the normal (1.00) or increased (1.67) P_up_ and the reduced g_Kr_ (0.48). Temporal behaviors of the model cells were computed for 30 min with pacing at 0.2 Hz; V_m_, Ca_SR_, Ca_i_, I_NCX_ and I_CaL_ for additional 30 s are shown as steady-state dynamics. The dots indicate spontaneous SR Ca^2+^ releases as evidenced by abrupt falls of Ca_SR_ and resulting increases in Ca_i_ and inward I_NCX_.

Following the finding of spontaneous SR Ca^2+^ releases which occurred especially under β-AS with enhanced I_CaL_ and SR Ca^2+^ uptake, we next examined how SR Ca^2+^ uptake/release (intracellular Ca^2+^ dynamics) and I_NCX_ regulated by the intracellular Ca^2+^, as well as I_CaL_ regulated by the subspace Ca^2+^, affect EAD formation and bifurcations of dynamical behaviors in the mTP06b model by changing P_up_, the scaling factor for I_NCX_, or g_CaL_. Figure 6A shows phase diagrams on the P_up_–g_Kr_ and P_up_–g_CaL_ parameter planes. P_up_ values were varied from zero to 2-times the control value, assuming the effects of SR Ca^2+^ pump inhibitors and β-AS (Maltsev and Lakatta, 2010; Briston et al., 2014). The region of EADs shrank with reducing P_up_ as in the K05 and O11 models (Kurata et al., 2017), while broadening at higher P_up_; however, for the emergence of EADs (red solid lines in Figure 6A), the critical g_Kr_ value was not decreased but slightly increased (the critical g_CaL_ value was not increased but slightly decreased) as P_up_ reduced. The facilitated EAD formation at smaller P_up_ was associated with increased Ca_i_ and resulting inward shift in I_NCX_ as well as slight increases in I_CaL_ during AP late phase 2 (see Supplementary Figure S6A). The two-parameter bifurcation analysis offered further information on how the region of EADs depends on P_up_ and g_Kr_. As shown in Supplementary Figure S7, the critical set of the emergence of EADs and the HB set were mostly parallel to the P_up_ and g_Kr_ axes, respectively, suggesting that alterations in P_up_ contributed not to EAD formation but to rather stability changes of EP (V_E__3_) in the non-paced mTP06b model; HB points disappeared with the emergence of spontaneous V_m_ and Ca^2+^ oscillations at higher P_up_, indicating that the SR Ca^2+^ uptake/release machinery destabilizes EPs and thereby induces spontaneous oscillations. When g_Kr_ was markedly reduced in the mTP06b model, spontaneous SR Ca^2+^ releases to cause transient increases in intracellular Ca^2+^ concentrations (Ca_ss_ and Ca_i_) and resulting activation of inward I_NCX_ occasionally occurred with prolonged APD (Figure 6B-a). Increasing P_up_ shortened the time to the emergence of the first spontaneous Ca^2+^ release and raised the incidence and frequency of spontaneous Ca^2+^ oscillations to yield EADs or V_m_ oscillations (Figure 6B-b).

Contributions of I_NCX_ to bifurcations and EAD formation were also explored in relation to those of intracellular Ca^2+^ dynamics, SR Ca^2+^ cycling, and I_CaL_. The scaling factor of I_NCX_ was varied from 0.1 to 10, within the range of experimental changes in Na^+^/Ca^2+^ exchanger densities or I_NCX_ (Milberg et al., 2008, 2012b; Pott et al., 2012). On the I_NCX_–g_Kr_ and I_NCX_–g_CaL_ parameter planes (Supplementary Figure S8), enhancement of I_NCX_ yielded the upward shift in the critical g_Kr_ and downward shift in the critical g_CaL_ for EAD formation (see red curves in Supplementary Figure S8). However, the TP06b model did not exhibit a significant shift in the critical g_Kr_ or g_CaL_ for EAD formation when I_NCX_ was reduced; only small (20–30%) inhibition of I_NCX_ was effective in shifting the critical points toward the prevention of EADs, with further inhibition resulting in the promotion of EADs. Whether EADs emerge or not depended mainly on the amplitude of inward I_NCX_ and I_CaL_ during the AP late phase 2: As exemplified in Supplementary Figure S6B, disappearance of EADs with lower I_NCX_ density was accompanied by a decrease of inward I_NCX_ and a slight reduction of I_CaL_ with increased inactivation during the preconditioning phase just before initiation of the first EAD (see the ellipses and inset in Supplementary Figure S6B).

We finally examined effects of I_CaL_ on EAD formation in the paced mTP06b model and bifurcations of dynamical behaviors in the non-paced mTP06b model by changing g_CaL_. g_CaL_-dependent changes in AP dynamics observed in the paced model cell when I_Ks_ was normal (g_Ks_ = 1) and one-parameter bifurcation diagrams as functions of g_CaL_ for the non-paced cell model are shown in Supplementary Figures S9A-i,ii, respectively. The one-parameter bifurcation diagrams for g_CaL_ (Supplementary Figure S9A-ii) suggest the scenario of EAD formation during enhancement of I_CaL_, which is different from those in the K05 and O11 models (Kurata et al., 2017): Increments of g_CaL_ yielded unstable EPs via a saddle-node bifurcation (SNB) of EPs and unstable LCs via a SNB of LCs. With normal I_Ks_ (g_Ks_ = 1), an enhanced g_CaL_ of 1.298-fold the control value was high enough for EAD formation in the mTP06b model (Supplementary Figure S9A-i), whereas unrealistically large increases in g_CaL_ (to 4.248-fold the control value) were required in the mTP06a model (Supplementary Figure S10A). Supplementary Figure S9B shows a phase diagram of AP behaviors in the paced model cell (Supplementary Figure S9B-i) and a two-parameter bifurcation diagram for the non-paced model cell (Supplementary Figure S9B-ii), as well as the merged diagram (Supplementary Figure S9B-iii), on the g_CaL_–g_Ks_ parameter planes. Decreasing g_Ks_ shifted the critical g_CaL_ value for EAD generation toward lower values and enlarged the g_CaL_ region of EADs (RF) in the mTP06b model. Larger g_CaL_ (going into the RF region in Supplementary Figures S9B-i,iii) led to the AP behavior classified into the RF type with small-amplitude spontaneous V_m_ oscillations around unstable LCs. EPs (V_E__3_) in the mTP06 models were unstable independent of g_CaL_ unless g_Ks_ was extremely low or high; no HB occurred for moderate variation of g_Ks_ value and consequently stability changes of the EP did not occur (see also Supplementary Figure S9B-ii). In the I_Ks_-eliminated mTP06a/b models (g_Ks_ = 0), an EP (V_E__3_) was stabilized via supercritical HBs at relatively small g_CaL_; stable LCs emerging from the HBs were immediately destabilized via a period-doubling bifurcation (PDB) or Neimark-Sacker bifurcation (NSB) (Supplementary Figure S10B). EAD did not occur at g_Ks_ = 0; larger I_CaL_ caused repolarization failure, in this case, local response.

### Dynamical Mechanisms for Initiation and Termination of EADs in the mTP06 Model

#### I_Ks_ Activation-Dependent Bifurcations of the Fast Subsystem Associated With EAD Formation

**FIGURE 7:**
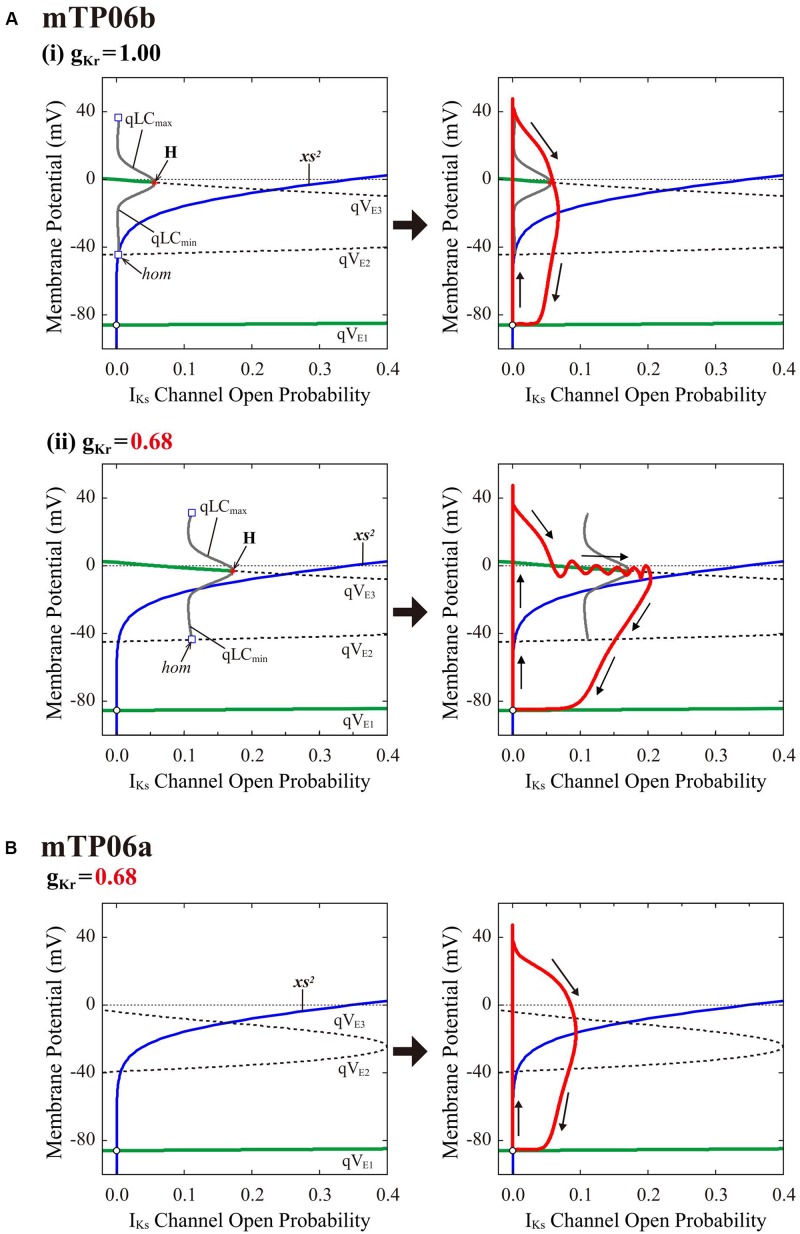
Dynamical mechanisms of EAD initiation and termination determined by the slow-fast decomposition analysis for the mTP06 models. Shown are one-parameter bifurcation diagrams of quasi-equilibrium points (qEPs) and quasi-limit cycles (qLCs), where the steady-state branches as loci of V_m_ at qEPs (qV_E__1__–__3_) and periodic branches as the potential minimum (qLC_min_) and maximum (qLC_max_) of qLCs are depicted as functions of the square of the I_Ks_ activation gating variable (*xs*^2^), i.e., I_Ks_ channel open probability for the fast subsystems of the g_Kr_-normal [**A-(i)**, *left*] and g_Kr_-reduced [**A-(ii)**, *left*] mTP06b model and g_Kr_-reduced mTP06a model (**B**, *left*). Other slow variables, Na_i_ and Ca_SR_, were fixed at constant values: Na_i_ = 6 mM for all cases; Ca_SR_ was fixed at the value which was reached just before occurrence of the first EAD or the maximum values during AP phase 2 (when no EAD occurred), i.e., at 0.5 mM and 1.5 mM for the normal and g_Kr_-reduced mTP06b model, respectively, and at 0.5 mM for the g_Kr_-reduced mTP06a model. The steady-state branches consist of the stable (green solid lines) and unstable (black dashed lines) segments. The periodic branches (gray solid lines) are all unstable. The blue lines indicate the steady-state *xs*^2^ curve. Trajectories of the full system (with the fixed Ca_SR_ and Na_i_) are superimposed on the bifurcation diagrams for the fast subsystems (red lines in each right panel). The arrows indicate the directions of changes in the state variables. H, Hopf bifurcation; *hom*, homoclinic bifurcation.

To clarify the dynamical mechanisms of EAD formation in the I_K__r_-reduced LQT2-type mTP06b model and why EADs emerge at larger g_Kr_ in the mTP06b model than in the mTP06a model (compare Figure 2 and Supplementary Figure S1), we further performed the *slow-fast decomposition analysis* (Tran et al., 2009; Qu et al., 2013; Xie et al., 2014). The I_Ks_ activation gating variable *xs* or I_Ks_ channel open probability (*xs*^2^) appears to be a slow variable yielding the termination of EADs (Figure 2A-a, *the second from top*). Thus, bifurcation diagrams for the fast subsystem composed of the state variables other than the slow variables *xs*, Na_i_ and Ca_SR_ were first constructed as functions of *xs*^2^, with Na_i_ and Ca_SR_ fixed at constant values (Figure 7, *left*); then, trajectories of the full system (with fixed Na_i_ and Ca_SR_) were superimposed on the diagrams (Figure 7, *right*). The quasi-EP (qEP), defined as a steady state of the fast subsystem, at depolarized quasi-V_m_ (qV_E__3_) has possessed a property of spiral sink in the mTP06b model (Figure 7A) but spiral source in the mTP06a model (Figure 7B) at *xs*^2^ = 0. Stable qEP in the former was destabilized via an HB as *xs*^2^ increased. The g_Kr_ reduction led to broadening of the *xs*^2^ region of stable qEPs (compare green traces of qV_E__3_ in Figures 7A-i,ii, *left*). The g_Kr_ reduction-induced broadening of the *xs*^2^ range of stable qEPs yielded a transient trapping of the full system trajectory in the attractor basin of the stable qEP (Figure 7A-ii, *right*). This trapping of the full system trajectory around the stable qEP as spiral sink sustained until the trajectory came across the steady-state *xs*^2^ curve. This trapping phenomenon was not observed in the mTP06a model (Figure 7B, *right*) or the I_Kr_-normal mTP06b model (Figure 7A-i, *right*), because the full system trajectories did not intersect with the stable steady-state branch (qV_E__3_) before intersecting the steady-state *xs*^2^ curve. These results indicate that an acceleration of the voltage-dependent I_CaL_ inactivation to form the mTP06b model from the mTP06a model plays a critical role in the stabilization of qV_E__3_, consequently leading to the trapping of the full system trajectory.

#### Dynamical Mechanisms of Spontaneous SR Ca^2+^ Release-Mediated EAD

**FIGURE 8:**
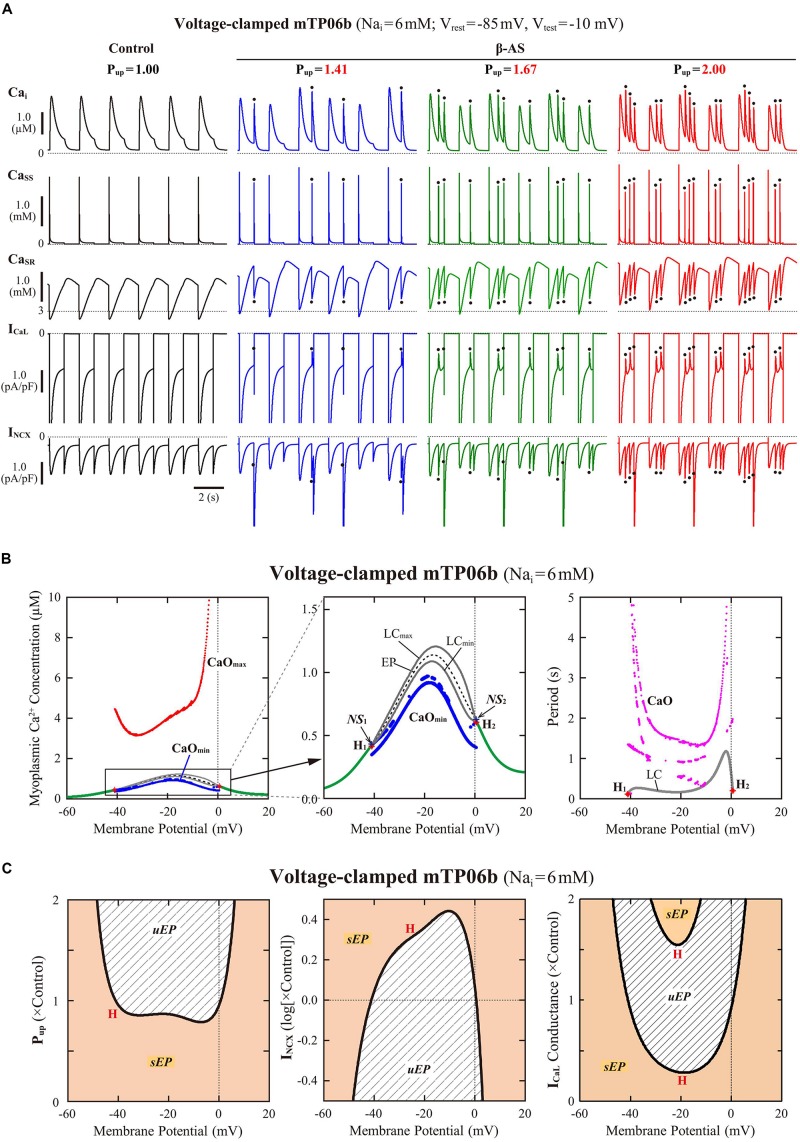
Stability and bifurcations of intracellular Ca^2+^ dynamics in voltage-clamped mTP06b model cell. **(A)** Dynamics of intracellular Ca^2+^ concentrations as well as Ca^2+^-dependent sarcolemmal currents in the Na_i_-fixed voltage-clamped model cell under the control condition (P_up_ = 1) and the conditions of β-AS (P_up_ = 1.41, 1.67, and 2.00). Temporal behaviors of the voltage-clamped model cell during a train of 1-s step depolarization from −85 mV to −10 mV at 0.5 Hz were computed for 10 min; Ca_i_, Ca_ss_, Ca_SR_, I_CaL_ and I_NCX_ for the last 12 s (6 pulses) are shown as steady-state dynamics. Spontaneous SR Ca^2+^ releases, as evidenced by abrupt falls of Ca_SR_ and increases in Ca_i_, Ca_ss_ and inward I_NCX_ with attenuated I_CaL_, occurred at higher P_up_ (indicated by the dots). **(B)** One-parameter bifurcation diagrams of the equilibrium point (EP) and extrema of limit cycles (LC_min/max_) and spontaneous Ca_i_ oscillations (CaO_min/max_) as functions of V_m_ for the voltage-clamped model cell (*left* and *middle*). The middle panel shows an enlarged diagram of the rectangular area in the left panel. The periods of spontaneous Ca^2+^ oscillations (CaO) and limit cycles (LC) are also plotted against V_m_ (*right*). **H_1__–__2_**, Hopf bifurcations of the EP; ***NS*_1__–__2_**, Neimark-Sacker bifurcations of the LC. **(C)** Two-parameter diagrams on the V_m_–P_up_ (*left*), V_m_–I_NCX_ (*middle*) and V_m_–g_CaL_ (*right*) planes, indicating how the unstable V_m_ range changed depending on P_up_, I_NCX_ and I_CaL_. HB values, i.e., the critical V_m_ at which an EP is (de)stabilized are plotted as functions of P_up_, I_NCX_ and I_CaL_ for the Na_i_-fixed mTP06b model; the HB points were very close to the Neimark-Sacker bifurcation points at which LCs were destabilized with the emergence of CaOs.

To clarify the dynamical mechanisms of spontaneous SR Ca^2+^ release-mediated EAD formation, we further examined the stability, dynamics and bifurcations of the voltage-clamped mTP06 model. Ca^2+^ dynamics during a train of 1-s depolarizing test pulses to −10 mV (from the holding potential of −85 mV) applied at 2-s intervals to mimic APs evoked by 0.5 Hz pacing were first determined for the mTP06b model with different P_up_ (Figure 8A). Spontaneous SR Ca^2+^ releases occurred when Ca_SR_ increased at higher P_up_, as indicated by the dots in Figure 8A; as P_up_ increased, the time to the first Ca^2+^ release and period of spontaneous Ca^2+^ releases shortened, and their frequency increased. Figure 8B shows one-parameter bifurcation diagrams of the steady-state stability and dynamics of Ca_i_ as functions of the clamped-V_m_ in the voltage-clamped mTP06b model. Steady-state intracellular Ca^2+^ concentrations (EPs) in the voltage-clamped model cell were stable at hyperpolarized and depolarized V_m_ (green traces in the right and middle panels of Figure 8B) but became unstable via supercritical HBs in the V_m_ range of AP phase 2 and early phase 3 (dashed traces in Figure 8B, *middle*). LCs emerging from the HB points were first stable but were destabilized via NSBs after small changes in V_m_; spontaneous Ca^2+^ oscillations occurred in the V_m_ range of unstable LCs, i.e., between *NS*_1_ and *NS*_2_ (gray traces labeled as LC_min_ and LC_max_ for the minimum and maximum Ca_i_ during LC oscillations in Figure 8B). As shown in Figure 8C, the unstable V_m_ region (*uEP*) was enlarged by increasing P_up_ (see Figure 8C, *left*), decreasing I_NCX_ activity (Figure 8C, *middle*), and/or enhancing I_CaL_ (Figure 8C, *right*), all of which led to increases in Ca_SR_.

**FIGURE 9:**
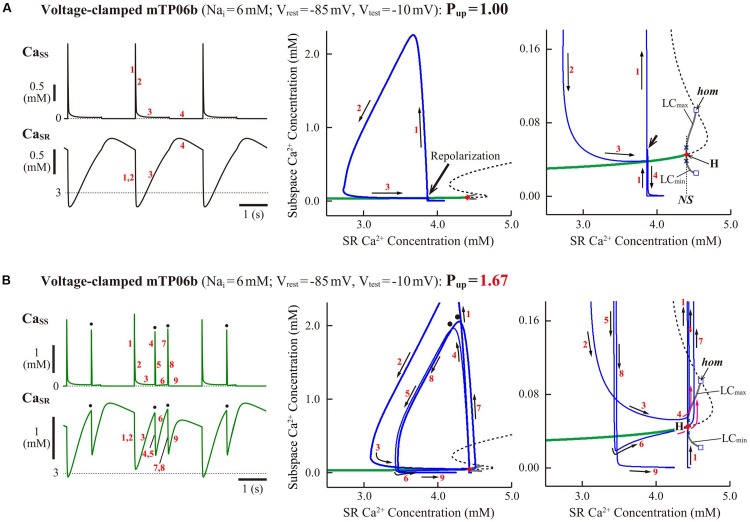
Slow-fast decomposition analysis for the slow variable Ca_SR_ of the voltage-clamped mTP06b model. Normal (*middle*) and expanded (*right*) scale views of one-parameter bifurcation diagrams, where the steady-state branch as a locus of Ca_ss_ at EPs, as well as periodic branches as the minimum (LC_min_) and maximum (LC_max_) of Ca_ss_ limit cycles (LCs) that were almost always unstable, are plotted as functions of Ca_SR_ for the fast subsystems of the voltage-clamped model cells with the normal **(A)** and increased **(B)** P_up_. The green solid and black dashed lines represent stable and unstable steady-state branch, respectively. Subcritical HB (H) with the emergence of an unstable LC, NSB from a stable LC to an unstable one following a SNB of LCs (*NS*), and homoclinic bifurcation (*hom*) are located. Trajectories projected onto the Ca_SR_-Ca_ss_ plane of the full system during a 1-s step depolarization to −10 mV (from −85 mV) and subsequent 1-s repolarization to −85 mV are superimposed on the diagrams; Ca_ss_ and Ca_SR_ dynamics in the full system during this voltage-clamp pulse are shown on the left. The labels “1”–“9” for the arrows on each diagram (*middle*, *right*) indicate the time course of changes in the state variables (Ca_ss_ and Ca_SR_) along the trajectories, with the labels also given for the Ca_ss_ and Ca_SR_ dynamics (*left*).

To further clarify the Ca_SR_-dependent mechanism of spontaneous SR Ca^2+^ releases in the P_up_-increased mTP06b model and why SR Ca^2+^ release-mediated EADs emerge more frequently at larger P_up_, we also performed the slow-fast decomposition analysis for the slow variable Ca_SR_. Bifurcation diagrams were constructed as functions of Ca_SR_ for the voltage-clamped fast subsystem composed of the voltage-independent state variables *f*_*CaL*_ (Ca^2+^-dependent inactivation gate for I_CaL_), *R* (proportion of closed SR Ca^2+^ release channels), Ca_ss_, and Ca_i_ (Figure 9). Trajectories of the voltage-clamped full system dynamics as shown in Figure 8A for the normal (1) and enhanced (1.67) P_up_ were superimposed on the diagrams. The steady states of the fast subsystem, stable at lower Ca_SR_ (green traces in the middle and right panels of Figure 9), became unstable via an HB at higher Ca_SR_ (dashed traces in the middle and right panels of Figure 9). In the P_up_-enhanced system, spontaneous SR Ca^2+^ releases as shown in blue trajectories in the middle and right panels of Figure 9B occurred when the full system trajectory, moving along the stable steady-state branch, passed through the HB point, i.e., when Ca_SR_ exceeded the HB value. In contrast, the P_up_-normal system did not exhibit spontaneous SR Ca^2+^ release, because an increment of Ca_SR_ (Ca^2+^ refilling of the SR) during Ca^2+^ transient decay was too slow for the full system trajectory to reach the HB point for Ca_SR_ before V_m_ repolarization (Figure 9A, *middle* and *right*).

## Discussion

In this study, we theoretically investigated dynamical mechanisms of EAD formation in the TP06 model for HVMs, which has often been used for simulations and theoretical analyses of reentrant arrhythmias, automaticity, multi-stability and EAD formation in HVMs, in relation to the model cell dynamical behaviors and their bifurcations. In summary, EAD formation and its dynamics in the paced (non-autonomous) mTP06 model cell basically depended on stability and bifurcations of the non-paced (autonomous) model cell. Bifurcation phenomena and dynamical mechanisms of EAD formation in the mTP06 model were different from those in the K05 and O11 models tested previously (Kurata et al., 2017) in several respects (see also Supplementary Materials for additional discussions).

### Validation of the mTP06 Model for EAD Reproducibility in LQT1 and LQT2 Conditions

#### EAD Formation in LQT1 and LQT2 Conditions (mTP06a vs. mTP06b)

Like the K05 model, the TP06b model with accelerated I_CaL_ inactivation could recapitulate EAD formation in the I_Ks_-reduced LQT1-type and I_Kr_-reduced LQT2-type HVMs. The mTP06b model was much more vulnerable to EAD formation than the mTP06a model, consistent with the previous experimental finding that slowing I_CaL_ inactivation eliminated EADs (Qu et al., 2013). As demonstrated by the slow-fast decomposition analysis (Figure 7), higher susceptibility of the mTP06b model to EAD development is attributable to the stabilization of qEPs at depolarized V_m_ close to the plateau V_m_ in the *xs*-parameterized fast subsystem by accelerating I_CaL_ inactivation.

EAD amplitudes in the mTP06b model (LQT1/2 versions) during pacing (∼30 mV) were smaller than those in the K05 and O11 models (Zimik et al., 2015; Kurata et al., 2017) as well as those in rabbit and guinea-pig ventricular myocyte models (Song et al., 2015; Zhong et al., 2018); however, they were comparable to those in many experimental reports for isolated HVMs (Verkerk et al., 2000; Veldkamp et al., 2001) and human iPS cell-derived LQT2 cardiomyocytes (Itzhaki et al., 2011) as well as for ferret, rabbit, and mouse ventricular myocytes (Marban et al., 1986; Liu et al., 2012; Edwards et al., 2014). Periods of EADs (∼200 ms) were shorter than those in the other HVM models, but comparable to the experimental data from HVMs (Verkerk et al., 2000).

#### Rate Dependence of EAD Formation (Validation for LQT2 Model)

In LQT2 patients, fatal cardiac events often occur during sleep or at rest, i.e., in bradycardia (Shimizu and Horie, 2011; Shimizu, 2013). The K05 and O11 models could partially reproduce the bradycardia-related EADs (Kurata et al., 2017). Like the other HVM models, the Na_i_-variable mTP06b model could partly reproduce the rate-dependent EAD generation in LQT2 patients (Figure 4). In the I_Kr_-reduced mTP06b model, however, EADs appeared only when pacing CLs increased to 3 s or more (i.e., pacing rates decreased to 20 beats/min or less) and extreme bradycardia continued for more than 4 min (Supplementary Figure S4). Although LQTS patients are known to exhibit sinus arrest or severe bradycardia due to coexisting sick sinus syndrome or atrio-ventricular block (Roden et al., 1996; Chiang and Roden, 2000), such long-lasting extreme bradycardia may be unlikely to occur often in LQT2 patients. Like the Luo-Rudy model for guinea-pig ventricular myocytes (Viswanathan and Rudy, 1999), the I_Kr_-reduced O11 model could reproduce pause-induced EAD formation on increasing a pacing CL from 1 s to 2 s, which was attributable to a decrease in I_Ks_ and Ca_i_ increase-mediated enhancement of inward I_NCX_ at the lower pacing rate (data not shown). In contrast, the mTP06 model did not exhibit EADs by a single pause or transient bradycardia, which may be a limitation of this model cell.

In our previous study for the Na_i_-variable K05 and O11 models (Kurata et al., 2017), the facilitation of EAD formation during lower rate pacing was accompanied by the decrease in Na_i_ and resulting reductions in outward I_NaK_ and inward shift of I_NCX_. This study also demonstrated for the mTP06b model that the facilitation of EAD formation at lower pacing rates was mainly due to the decrease in Na_i_ and resultant changes in I_NaK_ (Figure 4B). Thus, the major mechanism for bradycardia-related EADs in the mTP06b model is essentially the same as that in the K05 model. Bradycardia-induced EADs are believed to be ascribable to a reduction of I_Ks_ (and increment of I_CaL_). In the mTP06 model, however, I_Ks_ reduction (or I_CaL_ increment) did not occur when a pacing CL increased from 1 s to 2–5 s; deactivation of I_Ks_ was fast enough to complete before the next stimulus during 1-Hz pacing (Wang et al., 1994; Virág et al., 2001; Kurata et al., 2005; Jost et al., 2007).

#### EAD Formation During β-AS (Validation for LQT1 Model)

In LQT1 patients with smaller I_Ks_, fatal cardiac events are exercise-induced (tachycardia-related), because adrenergic enhancement of I_CaL_ is no longer counterbalanced by the concomitant stimulation of I_Ks_; the smaller increase in I_Ks_ leads to the occurrence of EADs that trigger ventricular tachyarrhythmia. The K05 model, but not the O11 model, could reproduce this I_Ks_ reduction-related EAD formation as a cause of ventricular tachycardia in LQT1 patients during β-AS (Sogo et al., 2016; Kurata et al., 2017). The mTP06b model was also capable of reproducing β-AS-related EAD formation in LQT1 cardiomyocytes, which could clearly be accounted for by the I_CaL_- and I_Ks_-dependent bifurcation properties of the model cell (Figure 5). I_CaL_/I_Ks_-dependent properties of EAD formation in the mTP06b model were very similar to those in the K05 model (Kurata et al., 2017), whereas I_CaL_/I_Ks_-dependent HB properties of the mTP06 models were totally different (Supplementary Figures S9B, S10). However, EAD formation in the mTP06b model during β-AS was different in a mechanism from that in the K05 model: EADs in the mTP06b model involved spontaneous SR Ca^2+^ releases and resulting increments of inward I_NCX_ (Figure 5A and Supplementary Figure S4), while those in the K05 model solely I_CaL_-reactivation dependent, not involving spontaneous SR Ca^2+^ releases (Kurata et al., 2017). It is likely that the spontaneous SR Ca^2+^ release is implicated in EAD formation during β-AS; delayed afterdepolarizations (DADs), known to be induced by spontaneous SR Ca^2+^ releases, often accompanied EADs (Priori and Corr, 1990; Volders et al., 2000; Zhao et al., 2012). Amplitudes of spontaneous SR Ca^2+^ releases in the mTP06b model appeared to be comparable to or slightly larger than those observed in experimental studies during β-AS (Volders et al., 2000; Zhao et al., 2012) or for LQT2 cardiomyocytes (Choi et al., 2002; Němec et al., 2010, 2016; Parikh et al., 2012) unless AP phase 2 was extremely long (Figure 8A and Supplementary Figure S5), while much larger than those reproduced by another HVM model (Trenor et al., 2013) and rabbit ventricular myocyte models (Milberg et al., 2012b; Song et al., 2015; Wilson et al., 2017; Zhong et al., 2018). More sophisticated HVM models incorporating β-AS-related modulating factors, like those developed by Saucerman et al. (2003) and Kuzumoto et al. (2008), are required for further investigations of the mechanisms of exercise-induced EAD formation in LQT1 HVMs.

### Comparisons With Other HVM Models for Bifurcation Phenomena and EAD Mechanisms

#### EAD Initiation Mechanisms (Roles of I_CaL_, I_NCX_, and SR Ca^2+^ Release)

At least two mechanisms appeared to underlie the initiation of phase-2 EADs in the mTP06b model: (1) I_CaL_ reactivation-dependent mechanism which operates and causes EADs even in the absence of spontaneous SR Ca^2+^ releases at lower P_up_ and lower pacing rates, and (2) spontaneous SR Ca^2+^ release-mediated mechanism activating inward I_NCX_ at higher P_up_ and higher pacing rates (Figures 2A-b, 6B). Coexistence of these two distinct mechanisms for EAD formation have been demonstrated experimentally as well (Zhao et al., 2012).

The major contribution of I_CaL_ to EAD formation was suggested in many previous experimental and theoretical studies for ventricular myocytes (January and Riddle, 1989; Ming et al., 1994; Guo D. et al., 2007; Yamada et al., 2008; Xie et al., 2010; Corrias et al., 2011; Madhvani et al., 2011; Chang et al., 2012a, b; Milberg et al., 2012a; Qu and Chung, 2012; Qu et al., 2013). EAD formation in the mTP06b model with lower P_up_ is also attributable to I_CaL_ reactivation in that reactivated I_CaL_ contributes to V_m_ depolarization (Figures 1C, 2A-a, and Supplementary Figure S6). In the K05 and O11 models, EADs often emerged in the vicinity of the critical point at which a stable LC appeared during I_CaL_ increases (Kurata et al., 2017), suggesting that EAD formation depends on I_CaL_ responsible for the instability of EPs and generation of stable LCs. EADs also occurred in the I_CaL_-enhanced mTP06b model when unstable LCs emerged, but stable LCs were not detected (Supplementary Figure S9A). The slow-fast decomposition analysis of the guinea-pig ventricular myocyte model have suggested that the I_CaL_-dependent destabilization of a qEP and formation of a stable quasi-LC (qLC) via an HB in the fast subsystem is required for EAD generation in the full system (Tran et al., 2009; Qu et al., 2013; Song et al., 2015). In the mTP06b model, however, transient trapping of the full system trajectory occurred around the stable and unstable qEPs without forming a stable qLC, indicating that the emergence of a stable qLC is not necessarily needed for EAD formation. This scenario for EAD formation is essentially the same as that in a two-current three-variable AP model (Xie et al., 2014). Nevertheless, the absence of a stable qLC may result in EADs of relatively small amplitudes, as demonstrated for the mTP06b model. As mentioned above, the initiation of EADs in the mTP06b model is attributable to the stabilization of qEPs at depolarized V_m_ in the *xs*-parameterized fast subsystem, which causes transient trapping of the full system trajectory around the stable qEP; I_Kr_ reduction promotes EAD formation by broadening the region of stable qEPs at depolarized V_m_ (Figure 7).

As another possible mechanism for EAD initiation, many recent experimental and simulation studies have strongly suggested the spontaneous SR Ca^2+^ release causing Ca_i_ oscillations, oscillatory increases in inward I_NCX_, and resulting V_m_ depolarization during β-AS (Choi et al., 2002; Volders et al., 2003; Zhao et al., 2012; Song et al., 2015; Wilson et al., 2017; Zhong et al., 2018) and in I_Kr_-reduced LQT2 cardiomyocytes (Choi et al., 2002; Kim et al., 2005; Němec et al., 2010, 2016), which is similar to the mechanism for DADs induced by spontaneous SR Ca^2+^ releases under Ca^2+^ overload conditions or β-AS (e.g., Volders et al., 2003; Zhao et al., 2012) and the Ca^2+^ clock mechanism for sinoatrial node cell pacemaking (Maltsev and Lakatta, 2009). The K05 or O11 model could not reproduce the spontaneous SR Ca^2+^ release as a cause of phase-2 EADs (Kurata et al., 2017). In contrast, the mTP06b model could clearly replicate this scenario in a Ca_SR_-dependent manner (Figures 2A-b, 5A, 6B, 9), while it was not found in the previous study using a modified TP06 model (Vandersickel et al., 2014). Such SR Ca^2+^ release-mediated EADs under β-AS conditions (Figure 5) have also been reproduced by the rabbit ventricular myocyte model (Volders et al., 2000; Song et al., 2015; Zhong et al., 2018).

One of the prominent properties of the mTP06 model is the instability of steady-state intracellular Ca^2+^ concentrations resulting in the spontaneous SR Ca^2+^ release at higher P_up_ to increase Ca_SR_ (Figures 8, 9). The K05 or O11 model did not exhibit spontaneous SR Ca^2+^ releases even at higher P_up_, because steady-state intracellular Ca^2+^ concentrations were always stable independently of V_m_; although Ca_i_ oscillations occurred during EADs in the K05 and O11 models, these Ca_i_ oscillations were not induced by spontaneous SR Ca^2+^ releases but by oscillatory reactivation of I_CaL_ (Kurata et al., 2017). Thus, this study newly suggests that the occurrence of spontaneous SR Ca^2+^ releases and Ca^2+^ oscillations as a cause of phase-2 EADs are attributable to instability of intracellular Ca^2+^ concentrations in a steady state, destabilization of which leads to spontaneous Ca^2+^ oscillations (Figures 8, 9). This scenario, i.e., steady-state destabilization for spontaneous Ca^2+^ oscillations involving ryanodine or IP_3_ receptors, has previously been suggested by bifurcation analyses for cardiac myocytes (Keizer and Levine, 1996; Tveito et al., 2012) and for other cells (Schuster et al., 2002; Higgins et al., 2006; Kusters et al., 2007). However, the Ca^2+^ oscillations reported in these previous studies were much longer in period than those observed in the mTP06b model, not relating to EAD formation. Wilson et al. (2017) demonstrated spontaneous SR Ca^2+^ releases and sustained Ca^2+^ oscillations in a voltage-clamped rabbit ventricular myocyte model, suggesting instability of intracellular Ca^2+^ dynamics; however, dynamical mechanisms for the Ca^2+^ oscillation were not clarified by bifurcation analysis. To the best of our knowledge, this is the first report demonstrating instability of steady-state intracellular Ca^2+^ concentrations and resulting spontaneous SR Ca^2+^ releases that cause EADs in the HVM model (Kurata et al., 2019). In the mTP06b model, enhanced I_CaL_ further contributed to spontaneous SR Ca^2+^ releases via the increment of SR Ca^2+^ contents and resultant enhancement of the instability of intracellular Ca^2+^ dynamics (Figure 8C). Elevations of Ca_ss_ by spontaneous SR Ca^2+^ releases caused transient I_CaL_ reductions due to Ca^2+^-dependent inactivation (Figures 2A-b, 6B, 8A), which may be regarded as a negative feedback mechanism leading to inhibition of EADs.

#### EAD Termination Mechanisms (Roles of I_Ks_, I_Kr_, and I_CaL_)

The mTP06 model requires I_Ks_ for EAD termination, i.e., repolarization failure occurred abruptly during I_Kr_ inhibition or I_CaL_ enhancement when I_Ks_ was absent or small, whereas I_Ks_ was not necessarily needed in the K05 or O11 model. Thus, EADs in the mTP06b model during pacing appeared to terminate in an I_Ks_ activation-dependent (or stimulus-dependent) manner: The open probability of I_Ks_ channels (*xs*^2^) increased progressively in the model cell with relatively small I_Kr_, i.e., LQT2-like cells (Figure 2A-a). Tran et al. (2009) suggested the major role of the slow I_Ks_ activation for the guinea-pig ventricular myocyte model by the slow-fast decomposition analysis in which the slow I_Ks_ activation gating variable was assumed to be a parameter for the fast subsystem. In the diagram for the slow gating variable-parameterized fast subsystem with a superimposed full system trajectory, gradual increases in the slow variable led the full system trajectory slowly across the stable steady-state branch of qEP and then into the region of the stable periodic branch of qLC through an HB point, resulting in the termination of EADs via a homoclinic bifurcation of qLC. This scenario is known as the Hopf-homoclinic bifurcation mechanism (Tran et al., 2009; Qu et al., 2013; Song et al., 2015; Huang et al., 2018). Consistent with these previous reports, the mTP06b model exhibited slow I_Ks_ activation-dependent EADs in the *xs*^2^ regions of stable and unstable qEPs (Figure 7A); however, a stable qLC region or homoclinic bifurcation to yield EAD termination was not detected for the fast subsystem of the mTP06b model. EAD terminated simply via the destabilization of a qEP in the I_Kr_-reduced mTP06b model, suggesting that the Hopf-homoclinic bifurcation scenario is not necessarily applicable.

In the I_Ks_-eliminated system, EADs would not terminate unless there exist other slow components or factors, such as the slowly inactivating I_CaL_ or late I_Na_ and intracellular Na^+^ accumulation to increase outward I_NaK_ gently. Our previous study using the K05 and O11 models indicated that EAD termination might occur in a slow I_CaL_ inactivation-dependent manner when I_Ks_ was relatively small (Figure 3 in Kurata et al., 2017). However, I_CaL_ inactivation-dependent EAD termination was not clearly detected in the TP06 model. Other candidates for slow variables to cause EAD termination include the slow inactivation of late I_Na_ (Horvath et al., 2013; Trenor et al., 2013; Asakura et al., 2014) not incorporated into the TP06 model and gradual increases in Na_i_ (Chang et al., 2012a; Xie et al., 2015). After cessation of pacing, the I_Kr_-reduced and/or I_CaL_-enhanced mTP06b model could exhibit long-term EAD bursts the termination of which was induced by slow elevation of Na_i_ and resulting enhancement of outward I_NaK_ (data not shown). This Na_i_-dependent mechanism has previously been demonstrated for a rabbit ventricular AP model as well (Chang et al., 2012a). Nevertheless, the slow Na_i_ elevation (intracellular Na^+^ accumulation) is unlikely as a termination mechanism for short-term EADs during pacing at 0.2–2 Hz in the mTP06b model.

As another termination mechanism, stimulus-induced repolarization was observed when APD became very long with stable AP phase 2 (Figures 2A-b, 3B-ii, 5, 6). In the mTP06b model, it was yielded mainly by the prolonged decrease (inactivation) of I_CaL_ due to its slow recovery and resulting outward shift in the total membrane current after the stimulus off. In terms of bifurcation theory, this phenomenon is related to bistability (co-existence of two stable EPs at resting V_m_ and depolarized V_m_ close to AP phase 2) and a transition between the two stable EPs by the stimulus; the transition occurs when following an application of the stimulus current a trajectory of a system starting from one stable EP at the depolarized V_m_ goes outside the attractor basin of the stable EP and enters the attractor basin of the other stable EP at the resting V_m_ (Vinet and Roberge, 1990). Bistability and the stimulus-induced transition between two stable states were found in other cardiomyocyte models (Landau et al., 1990; Vinet and Roberge, 1990). We could not find any experimental evidence for the AP repolarization induced by a stimulus current during stable AP phase 2, but it is theoretically possible. Experimental studies for wider ranges of channel conductance or other parameters may verify that the stimulus-induced repolarization really occurs.

### Limitations and Perspectives of Study

As summarized in our preceding article (Kurata et al., 2017), bifurcation analyses have been used for elucidating the dynamical mechanisms of sinoatrial node pacemaking, abnormal automaticity in ventricular myocytes, generation of biological pacemaker activity, and EAD formation in ventricular myocytes. These theoretical studies have clearly demonstrated the significance of bifurcation analyses for general understanding and systematic description of the dynamical mechanisms of normal and abnormal oscillatory behaviors.

There are many limitations of our study including incompleteness of the model and inconsistency between model predictions and experimental observations, as well as the lack of experimental evidence for bifurcation phenomena in real HVMs. The aim of this study was not to refine but to validate the TP06 model. Nevertheless, more sophisticated HVM models have to be used or developed for more detailed theoretical investigations. As mentioned above, simulated Ca^2+^ transients induced by spontaneous SR Ca^2+^ releases during AP phase 2 were larger than those observed in many experimental studies. The larger Ca^2+^ transients in the model cell may be due to the one compartment SR with weak Ca^2+^ leak, which results in higher SR Ca^2+^ load and greater Ca^2+^ releases during AP phase 2; a two-compartment SR model may be required for reproducing experimentally observed smaller Ca^2+^ releases, as suggested previously (Wilson et al., 2017). Moreover, incorporation of more elaborate schemes for the mechanisms of SR Ca^2+^ release and intra-SR Ca^2+^ transfer (Laver, 2007, 2009; Chen et al., 2014; Song et al., 2015; Zhong et al., 2018) would also be crucial. Our preceding (Kurata et al., 2017; Tsumoto et al., 2017) and present studies have demonstrated that EAD mechanisms are different depending on models and parameter values. Therefore, we have to test as many models as possible for providing more profound understanding of EAD mechanisms.

In this study, bifurcation analysis was limited to a single cell model. However, EAD-related arrhythmias are suggested to be induced by synchronization of EADs in multiple cells (Sato et al., 2009; Xie et al., 2010) and also influenced by heterogeneity of ventricular myocytes; because of electrotonic interactions, EAD formation in multicellular or tissue models including epicardial, endocardial and M cell models may be very different in conditions from that in single cell models (Gibb et al., 1994; Huelsing et al., 2000; Weiss et al., 2010; Corrias et al., 2011). Therefore, we need investigations of the mechanisms for EAD formation and for triggering arrhythmias in human ventricles *in vivo*, which require multicellular (tissue) models, like those used in previous simulation studies (Weiss et al., 2010; de Lange et al., 2012; Vandersickel et al., 2014; Chang et al., 2015; Liu et al., 2018). Despite many limitations, our studies provide significant insights into the dynamical mechanisms of EAD generation in LQT1 and LQT2 HVMs by utilizing recently developed HVM models.

## Author’s Note

This manuscript has been released as a Pre-Print at https://www.biorxiv.org/content/10.1101/613182v1 (Kurata et al., 2019).

## Data Availability Statement

All datasets generated for this study are included in the article/Supplementary Material.

## Author Contributions

YaK conception and design of the research and drafted the manuscript. YaK and KT performed the programing, simulations, bifurcation analyses, and analyzed the data. YaK, KT, and KH interpreted the results. YaK, KT, MT, and YuK prepared the figures. YaK, KT, KH, and IH edited and revised the manuscript. YaK, KT, KH, IH, MT, and YuK approved the final version of the manuscript.

## Conflict of Interest

The authors declare that the research was conducted in the absence of any commercial or financial relationships that could be construed as a potential conflict of interest.

## Footnotes

1 http://models.cellml.org/exposure/a7179d94365ff0c9c0e6eb7c6a787d3d

2 http://physiodesigner.org/

3 https://phdb.unit.oist.jp/modeldb/

4 http://www.physiodesigner.org/simulation/flint/
