# Supplementary material for: Multiple Dynamical Mechanisms of Phase-2 Early Afterdepolarizations in a Human Ventricular Myocyte Model: Involvement of Spontaneous SR Ca2+ Release
Source: Front Physiol. 2020 Jan 10;10:1545. doi: 10.3389/fphys.2019.01545 (PMC6965073; doi:10.3389/fphys.2019.01545)

Online Supplementary Materials

Multiple Dynamical Mechanisms of Phase-2 Early Afterdepolarizations in a Human Ventricular Myocyte Model: *Involvement of spontaneous SR Ca^{2+} release*

Yasutaka Kurata, Kunichika Tsumoto, Kenshi Hayashi, Ichiro Hisatome, Yuhichi Kuda, and Mamoru Tanida

1. Methods for Stability and Bifurcation Analyses

Basic methods

Changes in topological properties of solutions in ordinary differential equations, e.g., a change in the number of equilibrium points (EPs) or limit cycles (LCs), a change in the stability of an EP or LC, and a transition from a periodic to quiescent state, may be caused by altering parameters. This phenomenon is called *bifurcation* and the investigation of bifurcations depending on parameters is called *bifurcation analysis*. In physiological terms, EPs and LCs correspond to stationary states (resting states) and oscillatory states (spontaneous oscillations), respectively, and bifurcation phenomena that can be observed in cardiac cells include a cessation or generation of pacemaker activity and occurrence of arrhythmic dynamics (Landau *et al.* 1990; Vinet & Roberge, 1990; Guevara & Jongsma, 1992). For practical bifurcation analysis, we used 1) a MATLAB nonlinear equation solver (*fsolve*) implementing the Newton-Raphson algorithm to locate EPs and detect bifurcations of EPs; 2) MATLAB ODE solvers (*ode15s* and *ode45*) to calculate stable LCs and detect bifurcations of LCs; and 3) CL_MATCONT, a *continuation toolbox* for MATLAB (Dhooge *et al*. 2006), to locate EPs and LCs as well as to detect bifurcation points. The stability and types of bifurcations of EPs and LCs were determined by calculating eigenvalues of Jacobian matrices for EPs and characteristic multipliers for LCs (Parker & Chua, 1989; Tsumoto *et al.* 2012).

Bifurcations to occur in the mTP06 model were as follows: 1) Hopf bifurcation (HB) of EPs at which the stability of an EP reverses with emergence or disappearance of a LC; 2) saddle-node bifurcation (SNB) of EPs and LCs at which two EPs (steady-state branches) or two LCs (periodic branches) coalesce and disappear (or de novo creation of two EPs or periodic orbits occur); 3) period-doubling bifurcation (PDB) of LCs at which a stable period-K solution becomes unstable and spawns a stable period-2K solution; 4) Neimark-Sacker bifurcation (NSB) of LCs at which a stable LC becomes unstable with emergence of torus trajectories; and 5) homoclinic bifurcation (*hom*) of LCs at which a periodic solution is born from a homoclinic orbit that comes infinitely close to a single equilibrium point at infinity in time (Kuznetsov, 2003; Kurata *et al*. 2017).

Construction of one- and two-parameter bifurcation diagrams and phase diagrams

The information obtained from the bifurcation analysis enables us to know a parameter range over which the HVM model is in a stable stationary state or stable oscillatory state, the initial value dependency of the AP in the HVM model, and mechanisms of transitions between the stationary state and oscillatory one. A set of parameter values that cause bifurcations is called *bifurcation set* and a graph of these sets is called *bifurcation diagram*.

Non-paced model cells are quiescent at an EP (resting state and/or depolarized steady state) and/or exhibit a LC (spontaneous oscillation) depending on parameter values and initial conditions. For constructing one-parameter bifurcation diagrams, V_m values at EPs (V_E), potential minimum/maximum of LCs (LC_{min}/LC_{max}) and bifurcation points, as well as periods of LCs, were determined and plotted as functions of a given parameter. For construction of two-parameter bifurcation diagrams, the critical values of a first parameter for bifurcations to occur were determined by the one-parameter bifurcation analysis at each second parameter value. The sets of individual bifurcation points of the first parameter were plotted against the second parameter on a two-dimensional parameter plane.

In two-parameter phase diagrams, critical points of the first parameter at which a qualitative change in the AP of the paced cell model, e.g., a change from an AP without EAD to AP with EAD, first occurred were obtained by AP simulations utilizing the aforementioned numerical integration method. Then, we changed the second parameter value slightly and executed AP simulations again using the preceding results as the new initial conditions. These critical points collected as a parameter set were plotted on the first and second parameter plane.

Slow-fast decomposition analysis

Stability and bifurcations of a fast subsystem are determined as functions of a slow variable by the slow-fast decomposition analysis (Doi *et al*. 2001; Tran *et al*. 2009; Qu *et al*. 2013; Xie *et al*. 2014; Kügler *et al*. 2018). This method can define EAD formation as transient trapping of a trajectory of the full system into the quasi-attractor, i.e., quasi-EPs (qEPs) and/or quasi-LCs (qLCs), transiently emerged in the fast subsystem (Qu *et al*. 2013). The gating variable for I_{Ks} activation (***xs***) and Ca^{2+} concentration in the SR (Ca_{SR}) were chosen as slow variables; bifurcation diagrams consisting of steady-state branches (for qEPs) and periodic branches (for qLCs) for the fast subsystem were constructed as functions of a slow variable (xs^2 or Ca_{SR}), with trajectories of the full system superimposed on the diagrams.

2. Additional Discussion

Comparisons with Other HVM Models for Effects of Modulating SR Ca^{2+} Cycling and I_{NCX}

The effects on EAD formation of modulating SR Ca^{2+} uptake/release in the mTP06 model were different from those in the other HVM models (Kurata *et al*. 2017). With respect to the onset of EADs during I_{Kr} inhibition or I_{CaL} enhancement, our previous study using the K05 model showed that enhancing SR Ca^{2+} uptake/release facilitated EAD generation via an inward shift of I_{NCX} and reduction of I_{CaL} which lowered the plateau V_m and thereby limited I_{Ks} activation during the preconditioning phase before EAD formation (Kurata *et al*. 2017). In the mTP06 model, however,

the effects of modulating SR Ca^{2+} uptake/release (changing P_{up}) on the onset of EADs were opposite to those in the K05 model, while similar to those in the O11 model; inhibition of SR Ca^{2+} uptake/release or Ca^{2+} transient did not prevent but slightly facilitated I_{CaL} reactivation-dependent EAD formation during I_{Kr} inhibition or I_{CaL} increments (**Figure 6A**). This inconsistency is mainly due to the differences in SR Ca^{2+} uptake/release mechanisms and Ca_i-dependent behaviors of I_{CaL} and I_{NCX}. The effects on EP stability in the three HVM models were completely different: SR Ca^{2+} cycling destabilized EPs and induced spontaneous oscillations in the mTP06 model (**Figure 6** and Supplementary **Figure S7**), exerted no effect on EP stability (and little affected LC oscillation) in the K05 model, and broadened the parameter regions of stable EPs in the O11 model. Thus, the influences of SR Ca^{2+} cycling on bifurcations and EAD formation are model dependent; further studies using more sophisticated HVM models as well as experimental verifications are required.

In previous experimental studies, enhancing I_{NCX} tended to promote EADs (Pott *et al*. 2012), while inhibition of I_{NCX} prevented EADs (Nagy *et al*. 2004; Milberg *et al*. 2008; Milberg, Pott, *et al*. 2012; Zhao *et al.* 2012). The mTP06 model predicted facilitation of EAD formation by enhanced I_{NCX}, qualitatively consistent with the experimental finding (Pott *et al*. 2012) and our previous report for the K05 and O11 models (Kurata et al. 2017). This promotion of EAD generation was not only via enhancing inward I_{NCX} but also via secondary reductions in I_{CaL} inactivation and resultant increases of I_{CaL} window currents, as in the K05 and O11 models (Kurata *et al*. 2017). In the mTP06 model, small inhibition of I_{NCX} yielded the suppression of phase-2 EADs by primary reductions of inward I_{NCX} and secondary decreases of I_{CaL}, as in the other HVM models. However, greater inhibition of I_{NCX} led to facilitated EAD formation, inconsistent with experimental reports (Milberg *et al.* 2008; Milberg, Pott, *et al*. 2012) and our previous report for the other HVM models (Kurata *et al.* 2017). Thus, influences of I_{NCX} modifications were also model dependent; more quantitative experimental verifications and theoretical studies using more elaborate HVM models are needed.

3. Supplementary References

Dhooge, A., Govaerts, W., Kuznetsov, Yu.A., Mestrom, W., Riet, A.M., et al. (2006). MATCONT and CL_MATCONT: Continuation toolboxes in MATLAB. Published on the web (http://www.matcont.ugent.be/matcont.html).

Doi, S., Nabetani, S., and Kumagai, S. (2001). Complex nonlinear dynamics of the Hodgkin-Huxley equations induced by time scale changes. *Biol. Cybern.* **85**, 51–64. doi: 10.1007/PL00007996

Guevara, M.R., and Jongsma, H.J. (1992). Three ways of abolishing automaticity in sinoatrial node: ionic modeling and nonlinear dynamics. *Am. J. Physiol.* **262**, H1268–H1286. doi: 10.1152/ajpheart.1992.262.4.H1268

Kügler, P., Erhardt, A.H., and Bulelzai, M.A.K. (2018). Early afterdepolarizations in cardiac action potentials as mixed mode oscillations due to a folded node singularity. PLoS One **13**, e0209498. doi: 10.1371/journal.pone.0209498

Kurata, Y., Tsumoto, K., Hayashi, K., Hisatome, I., Tanida, M., Kuda, Y., et al. (2017). Dynamical mechanisms of phase-2 early afterdepolarizations in human ventricular myocytes: insights from bifurcation analyses of two mathematical models. *Am. J. Physiol. Heart Circ. Physiol.* **312**, H106–H127. doi: 10.1152/ajpheart.00115.2016

Kuznetsov, Y.A. (2003). Elements of applied bifurcation theory (3rd Ed). Springer-Verlag, New York.

Landau, M., Lorente, P., Michaels, D., and Jalife, J. (1990). Bistabilities and annihilation phenomena in electrophysiological cardiac models. *Circ. Res.* **66**, 1658–1672.

Milberg, P., Pott, C., Fink, M., Frommeyer, G., Matsuda, T., Baba, A., et al. (2008). Inhibition of the Na^+/Ca^{2+} exchanger suppresses torsades de pointes in an intact heart model of long QT syndrome-2 and long QT syndrome-3. *Heart Rhythm* **5**, 1444–1452. doi: 10.1016/j.hrthm.2008.06.017

Milberg, P., Pott, C., Frommeyer, G., Fink, M., Ruhe, M., Matsuda, T., et al. (2012). Acute inhibition of the Na^{+}/Ca^{2+} exchanger reduces proarrhythmia in an experimental model of chronic heart failure. *Heart Rhythm* **9**, 570–578. doi: 10.1016/j.hrthm.2011.11.004

Nagy, Z.A., Virág, L., Tóth, A., Biliczki, P., Acsai, K., Bányász, T., et al. (2004). Selective inhibition of sodium–calcium exchanger by SEA-0400 decreases early and delayed afterdepolarization in canine heart. *Br. J. Pharmacol.* **143**, 827–831. doi: 10.1038/sj.bjp.0706026

Parker, T.S., and Chua, L.O. (1989). Practical numerical algorithms for chaotic systems. Springer-Verlag, New York.

Pott, C., Muszynski, A., Ruhe, M., Bögeholz, N., Schulte, J.S., Milberg, P., et al. (2012). Proarrhythmia in a non-failing murine model of cardiac-specific Na^{+}/Ca^{2+} exchanger overexpression: whole heart and cellular mechanisms. *Basic Res. Cardiol.* **107**, 1–13. doi: 10.1007/s00395-012-0247-7

Qu, Z., Xie, L.H., Olcese, R., Karagueuzian, H.S., Chen, P.S., Garfinkel, A., et al. (2013). Early afterdepolarizations in cardiac myocytes: beyond reduced repolarization reserve. *Cardiovasc. Res.* **99**, 6–15. doi: 10.1093/cvr/cvt104

Tran, D.X., Sato, D., Yochelis, A., Weiss, J.N., Garfinkel, A., and Qu, Z. (2009). Bifurcation and chaos in a model of cardiac early afterdepolarizations. *Phys. Rev. Lett.* **102**, 258103. doi: 10.1103/PhysRevLett.102.258103

Tsumoto, K., Ueta, T., Yoshinaga, T., and Kawakami, H. (2012). Bifurcation analyses of nonlinear dynamical systems: From theory to numerical computations. *NOLTA.* **3**, 459 − 476. doi: 10.1587/nolta.3.458

Vinet, A., and Roberge, F.A. (1990). A model study of stability and oscillations in the myocardial cell membrane. *J. Theor. Biol.* **147**, 377–412. doi: 10.1016/s0022-5193(05)80495-3

Xie, Y., Izu, L.T., Bers, D.M., and Sato, D. (2014). Arrhythmogenic transient dynamics in cardiac myocytes. *Biophys. J.* **106**, 1391–1397. doi: 10.1016/j.bpj.2013.12.050

Zhao, Z., Wen, H., Fefelova, N., Allen, C., Baba, A., Matsuda, T., et al. (2012). Revisiting the ionic mechanisms of early afterdepolarizations in cardiomyocytes: predominant by Ca waves or Ca currents? *Am. J. Physiol. Heart Circ. Physiol.* **302**, H1636–H1644. doi: 10.1152/ajpheart.00742.2011

4. Supplementary Tables

Table S1: Parameter values for the M cell versions of the mTP06a and mTP06b models relative to those for the original TP06 model.

Parameters	Units	Original TP06	mTP06a	mTP06b
Maximum conductance of I_{CaL} (g_{CaL})	cm^3/ms/μF	0.0000000398	0.0000000398	0.0000000796
Time constant of voltage-dependent I_{CaL} inactivation	(ratio)	1	1	0.5
Maximum conductance of I_{Kr} (g_{Kr})	nS/pF	0.153	0.2295	0.2295
Maximum conductance of I_{Ks} (g_{Ks})	nS/pF	0.098	0.0392	0.0392
Maximum rate of Ca^{2+} uptake to SR	mM/ms	0.006375	0.003825	0.003825

Table S2: Modifications of parameters for the conditions of β-adrenergic stimulation (β-AS)

Parameters	Units	Control (mTP06b)	β-AS (*ratio)
Maximum conductance of I_{CaL} (g_{CaL})	cm^3/ms/μF	0.0000000796	1.4–1.8*
Maximum conductance of I_{Ks} (g_{Ks})	nS/pF	0.0392	2.0*
Voltage shift in I_{Ks} activation gate	mV	0	−8.0
Maximum Na^+-K^+ pump current (I_{NaK})	pA/pF	2.724	1.20*
Maximum rate of Ca^{2+} uptake to SR	mM/ms	0.003825	1.41*

Table S3: Initial conditions of state variables for computing dynamic behaviors of the M cell versions of the original TP06, mTP06a and mTP06b models as shown in Figure 1.

Variables	Definitions	Units	Original TP06	mTP06a	mTP06b
V	Membrane potential (mV)	mV	-86.596524	-86.596551	-86.580995
m	Activation gating variable for I_{Na}		0.00128231	0.00128230·	0.00128657
h	Inactivation gating variable for I_{Na}		0.78040927	0.78040994	0.78002868
j	Recovery gating variable for I_{Na}		0.78040927	0.78040994	0.78002868
d_L	Activation gating variable for I_{CaL}		0.0000281050	0.0000281049·	0.0000281632
f_L	V_m-dependent inactivation gating variable for I_{CaL}		0.99992618	0.99992618	0.99992601
f_{L2}	2nd V_m-dependent inactivation gating variable for I_{CaL}		0.99957865	0.99957865	0.99957771
f_{Ca}	Ca^{2+}-dependent inactivation gating variable for I_{CaL}		0.99999826	0.99999826	0.99999572
r	Activation gating variable for I_{to}		0.0000000192435	0.0000000192435	0.0000000192934
s	Inactivation gating variable for I_{to}		0.99999836	0.99999836	0.99999835
xr_1	1st activation gating variable for I_{Kr}		0.000173937	0.000173935·	0.000174323
xr_2	2nd activation gating variable for I_{Kr}		0.48538462	0.48538491	0.48522301
xs	Activation gating variable for I_{Ks}		0.00293437	0.00293437·	0.00293762
R_prime	Activation gating variable for SR Ca^{2+} release channel		0.998160785	0.998116636	0.99704737
Ca_{ss}	Subspace Ca^{2+} concentration	mM	0.0000850477	0.0000850454·	0.000133586
Ca_i	Cytoplasmic Ca^{2+} concentration	mM	0.0000372687	0.0000372687·	0.0000378481
Ca_{SR}	Ca^{2+} concentration in SR	mM	0.385019	0.231026	0.238101237
Na_i	Intracellular Na^+ concentration	mM	10.3550	10.3550	10.3550
K_i	Intracellular K^+ concentration	mM	140	140	140

5. Supplementary Figure Legends

Supplementary Figure S1: I_{Kr}-dependent EAD generations and bifurcations in the I_{Ks}-normal and I_{Ks}-eliminated mTP06a/b models.

Potential extrema of simulated action potential (AP) and EADs, and the AP duration (APD) measured at 90% repolarization (APD_{90}) are plotted as functions of normalized g_{Kr} for the I_{Ks}-normal mTP06a **(A)** and I_{Ks}-removed mTP06a/b **(B)** model cells paced at 0.2 Hz **(i)**. AP dynamics were computed for 1 min at each g_{Kr} value, which was reduced from 1.0 to −0.2 or 1.2 to 0 at an interval of 0.001. The minimum V_m during AP phase 4 (V_{min}) and the maximum V_m during AP phase 2 before EAD formation (V_{max}) are represented by black dots for rhythmic APs, and by orange (V_{min}) and light green (V_{max}) dots for arrhythmic APs. When EADs appeared, their local potential minimum (EAD_{min}) and maximum (EAD_{max}) were plotted by blue and red dots, respectively. In the diagram for APD_{90}, the black, blue and magenta dots represent APD_{90} values for regular APs without EAD, regular APs with EADs, and arrhythmic APs with EADs, respectively (no-EAD: APs without EAD, +EAD: APs with EADs). One-parameter bifurcation diagrams with the steady-state branches as loci of V_m at equilibrium points ($V_{E1\text{-}3}$) and periodic branches as the potential minimum (LC_{min}) and maximum (LC_{max}) of limit cycles (LCs) as well as the period of LCs plotted against g_{Kr} are also shown for the non-paced mTP06a/b model cells **(ii)**. The steady-state branches consist of the stable (green solid lines) and unstable (black dashed lines) segments; the periodic branches are almost always unstable, while stable between the Hopf bifurcation (H) and Neimark-Sacker bifurcation (NS) points in Panel A-(ii). The inset in Panel B-(ii) for the I_{Ks}-removed mTP06b model is the expanded scale view of the vicinity of the Hopf bifurcation point (rectangular area). Representations and symbols are the same as in Figure 2B. ***SN***, saddle-node bifurcation of LCs.

Supplementary Figure S2: I_{Kr}/I_{Ks}-dependent EAD generations and bifurcations in the mTP06a model.

(**A**) A phase diagram indicating the region of EAD formation (and local responses) in the paced model cell **(i)** and two-parameter bifurcation diagrams for the non-paced model cell **(ii)** on the g_{Ks}–g_{Kr} parameter plane. The panel **(iii)** is the diagram for which the phase diagram (i) is superimposed upon the two-parameter bifurcation diagram (ii). In the diagram for the paced model cell (i), the thick red solid, black dashed and thin black solid lines respectively indicate parameter sets of critical points at which short-term EADs (fast repolarization type of APs with EADs), long-term or sustained EADs (repolarization failure type of APs with EADs), and local responses emerged; parameter regions in which short-term EADs, long-term or sustained EADs, and local responses can be observed are shown as the light gray (fR), blue (RF) and dotted regions, respectively. In the diagram for the non-paced model cell (ii), **H**, **SO$_1$**, and **SO$_2$** indicate parameter sets of Hopf bifurcation points, critical points at which SOs emerged, and critical points at which SOs switched into quiescence, respectively. The parameter region in which convergence to the steady-state (V_{E3}) can occur is denoted as the orange region. The labels "*sEP*" and "*uEP*" indicate the areas of stable and unstable equilibrium points (EPs), respectively, divided by the Hopf bifurcation curves (H). The points labeled as "N", "*LQT1*" and "*LQT2*" denote the normal, LQT1, and LQT2 condition, respectively. The asterisks in the panels (i) and (iii) indicate the condition under which the paced model cell behaved as shown in Panel B.

(B) Simulated behaviors of APs, sarcolemmal ionic currents (I_{Ks}, I_{CaL}, I_{NCX}) and intracellular Ca^{2+} concentrations (Ca_i, Ca_{SS}, Ca_{SR}) during 0.2-Hz pacing in the model cell with the parameter set as indicated by the asterisks in Panel A. Temporal behaviors of the variables were computed for 30 min; those for 10 s elicited by additional 2 stimuli are shown.

Supplementary Figure S3: Rate dependence of EAD generation in the mTP06b model.

The phase diagrams indicating the region of EAD formation (and local responses) for the paced model cell are superimposed on the two-parameter bifurcation diagrams for the non-paced model cell on the g_{Ks}–g_{Kr} parameter plane. The Na_i-variable **(A)** and Na_i-fixed **(B)** versions were tested for comparison. The pacing cycle lengths (CLs) were set to 5 s (*left*) and 1 s (*right*). The thick red solid, black dashed and thin black solid lines represent the parameter sets of critical points for occurrences of short-term EADs (APD_{90} < 5 s), long-term or sustained EADs (APD_{90} > 5 s) and local responses, respectively; parameter regions of short-term EADs, long-term or sustained EADs and local responses are shown as the light-gray area labeled as "**fR**" (fast repolarization), blue area labeled as "**RF**" (repolarization failure) and dotted area, respectively. **H**, **SO$_1$**, and **SO$_2$** indicate parameter sets of Hopf bifurcation points, critical points at which SOs emerged, and critical points at which SOs switched into quiescence, respectively; parameter regions in which SOs and convergence to the steady state (V_{E3}), i.e., arrest, can be observed are shown as the shaded and orange regions, respectively. The points labeled as "N" denote the normal condition.

Supplementary Figure S4: Time course of APD changes and timing of EAD generation in the Na_i-variable g_{Kr}-reduced mTP06b model after slowing of pacing.

(A) Simulated AP dynamics of the model cell with g_{Kr} = 0.721 when a pacing CL (PCL) was increased from 1 s to 3 s. Temporal behaviors of the model cell paced at 1 Hz (CL = 1 s) were computed for 30 min to determine steady-state dynamics at 1 Hz, and then the pacing CL was abruptly increased to 3 s. Shown are an AP evoked by an additional (1801th) stimulus after 30-min pacing at 1 Hz, and those evoked by the 1st, 170th and 171th pacing stimuli with CL = 3 s.

(B) Temporal behaviors of the model cell when a pacing CL (PCL) was increased from 1 s to 3 s. APs elicited by the 4 stimuli, as well as changes in Ca_i, Ca_{SR}, Na_i, I_{CaL}, I_{Ks}, I_{NaK} and I_{NCX} during the APs, are shown to clearly demonstrate their time-series behaviors during the slower pacing.

Supplementary Figure S5: Simulated temporal behaviors of Ca_i in the I_{Ks}-normal and I_{Ks}-reduced (LQT1-type) mTP06b model cells under the basal and β-AS conditions.

Model cells were paced at 1 Hz for 30 min under the normal and β-AS conditions as indicated by the points and arrows in Figure 5B. The dots represent Ca_i elevations evoked by spontaneous SR Ca^{2+} releases.

Supplementary Figure S6: Influences of P_{up} and I_{NCX} on EAD formation in the mTP06b model with an increased g_{CaL}.

Simulated dynamics of the model cells with various P_{up} **(A)** or I_{NCX} **(B)** values and an increased g_{CaL} (1.31) are shown. P_{up} values were 0 (red), 1.0 (black) and 2.0 (blue) times the control value. I_{NCX} values were 0.67 (red), 1.0 (black) and 1.5 (blue) times the control value. Temporal behaviors of the model cells were computed for 30 min with pacing at 0.2 Hz; the responses to an extra stimulus of V_m, Ca_i, I_{NCX}, and I_{CaL} for 1.6 s are shown as steady-state dynamics. The dashed ellipses are to focus on the differences in I_{NCX} and I_{CaL} during the preconditioning phase just before initiation of the first EAD. The inset in the I_{CaL} window shows an expanded scale view of I_{CaL} behaviors in the ellipse. The vertical arrows indicate the changes with decreasing P_{up} (A) or I_{NCX} (B).

Supplementary Figure S7: AP behaviors and bifurcations on the P_{up}–g_{Kr} **(A)** and P_{up}–g_{CaL} **(B)** parameter planes in the mTP06b model.

Shown are phase diagrams depicting displacements of critical points for occurrences of short-term EADs (red solid lines), long-term or sustained EADs (black dashed lines) and local responses (black solid lines) in the paced model cell **(i)**, and two-parameter bifurcation diagrams with Hopf (H) and saddle-node (SN) bifurcation curves, and the critical curves for the occurrence (SO_1) and disappearance (SO_2) of SOs for the non-paced model cell **(ii)**, as well as the merged diagrams **(iii)**

for which the phase diagrams are superimposed on the two-parameter bifurcation diagrams. Representations and symbols are the same as in Figure 3A and Supplementary Figure S3.

Supplementary Figure S8: AP behaviors and bifurcations on the I_{NCX}–g_{Kr} **(A)** and I_{NCX}–g_{CaL} **(B)** parameter planes in the mTP06b model.

Shown are phase diagrams depicting displacements of critical points for occurrences of short term EADs (red solid lines) and long-term or sustained EADs (black dashed lines) in the paced model cells **(i)** and two-parameter bifurcation diagrams with the Hopf (H) or saddle-node (SN) bifurcation curve and the critical curve for the occurrence of SOs (SO_1) for the non-paced model cell **(ii)**, as well as the merged diagrams **(iii)** of the phase diagram (i) superimposed on the two-parameter bifurcation diagram (ii). I_{NCX} density is given as the common logarithm of ratios to the control value. Representations and symbols are the same as in Figure 3A and Supplementary Figure S3.

Supplementary Figure S9: I_{CaL}–dependent EAD generation and bifurcations in the mTP06b models.

(A) Potential extrema of simulated AP and EAD behaviors and APD_{90} for the paced model cell **(i)**, as well as one-parameter bifurcation diagrams with steady-state V_m ($V_{E1–3}$), potential extrema of LCs and SOs, and the periods of LCs and SOs for the non-paced model cell **(ii)**, plotted as functions of normalized g_{CaL}. Representations and symbols are the same as in Figure 2B and Supplementary Figure S1.

(B) A phase diagram **(i)** for the paced model cell, a two-parameter bifurcation diagram **(ii)** for the non-paced model cell, and the merged diagram for which the phase diagram is superimposed upon the two-parameter bifurcation diagram on the g_{CaL}–g_{Ks} parameter plane, depicting displacements of critical points at which EADs and local responses emerged, HB points (H), SNB points where unstable V_{E2} and V_{E3} coalesced and vanished (SN), critical points at which SOs emerged (SO_1) or

switched into quiescence (SO_2), and critical points at which repolarization failure occurred. In the panel **(i)**, the g_{CaL}–g_{Ks} parameter plane is divided into the areas of APs without EAD, APs with EADs repolarization of which occurred within 5 s after the last stimulus (fR), and repolarization failure (RF), as in Fig. 3A-(i). The points labeled as "N" denote the normal condition. In the panel **(ii)**, SOs appeared in the shaded region surrounded by SO_1, SO_2 and H curves.

Supplementary Figure S10: I_{CaL}–dependent EAD generations and bifurcations in the I_{Ks}-normal and I_{Ks}-eliminated mTP06a/b models.

Potential extrema of simulated APs and EADs, and APD_{90} are plotted as functions of normalized g_{CaL} for the I_{Ks}-normal mTP06a **(A)** and I_{Ks}-removed mTP06a/b **(B)** model cells paced at 0.2 Hz **(i)**. AP dynamics during 0.2-Hz pacing were computed for 1 min at each g_{CaL} value, which was increased from 0 to 2.5 or 5.0 at an interval of 0.002. The black dots indicate V_{min} and V_{max} for rhythmic APs, while orange and light green dots in Panel A indicate V_{min} and V_{max}, respectively, for arrhythmic APs. EAD_{min} and EAD_{max} were plotted by blue and red dots, respectively. In the diagram for APD_{90}, the black dots represent APD_{90} values for regular APs without EAD (no-EAD). One-parameter bifurcation diagrams depicting steady-state V_m at EPs ($V_{E1\text{-}3}$) and potential extrema of LCs ($LC_{min/max}$), and the period of LCs plotted against g_{CaL} are also shown for the non-paced mTP06a/b model cells **(ii)**. The steady-state branches ($V_{E1\text{-}3}$) consist of the stable (green solid lines) and unstable (black dashed lines) segments; the periodic branches are almost always unstable, while stable between the Hopf (H) and Neimark-Sacker (*NS*) or period-doubling (*PD*) bifurcation points.

Supplementary Figure S1

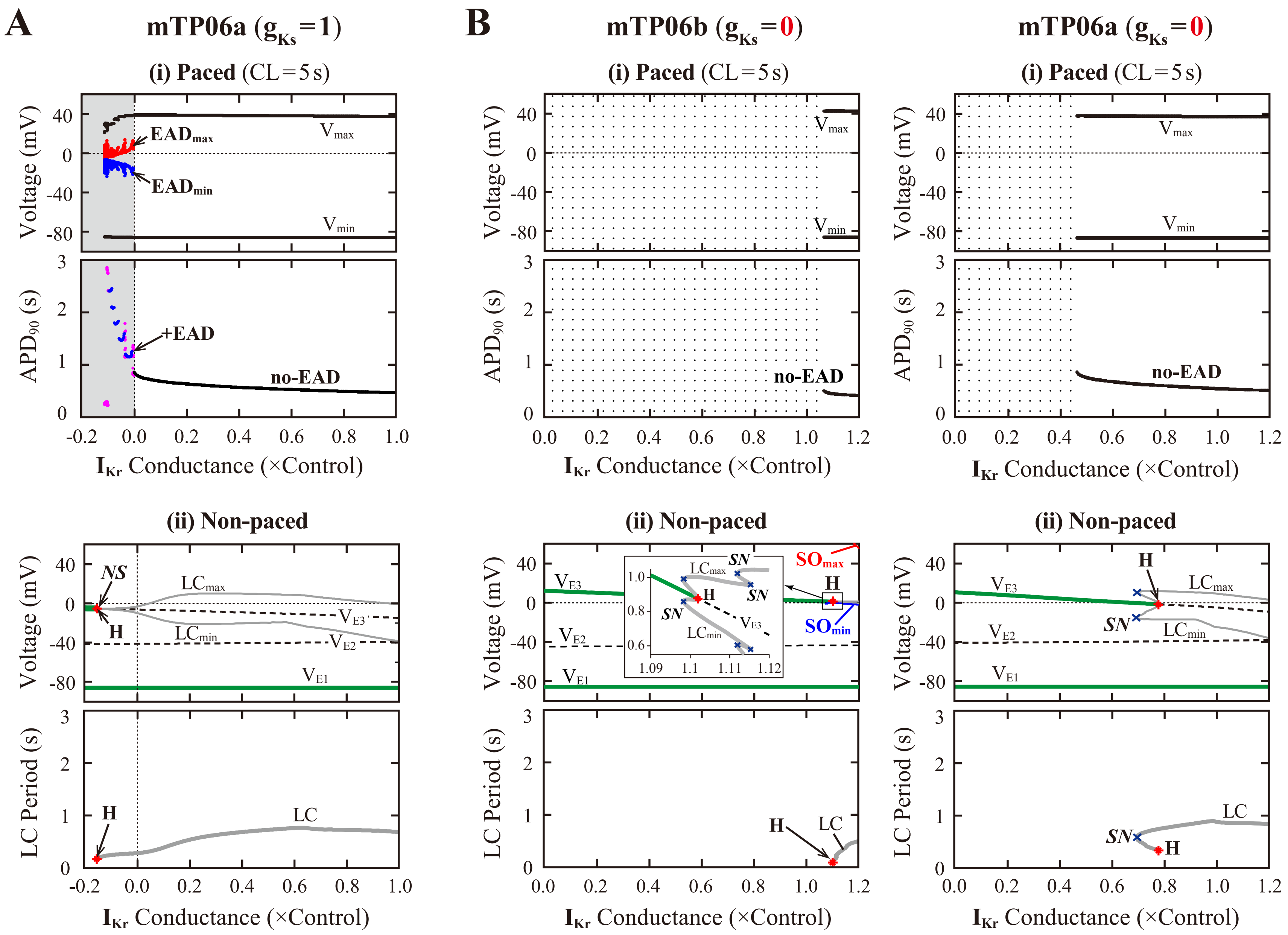

Supplementary Figure S2

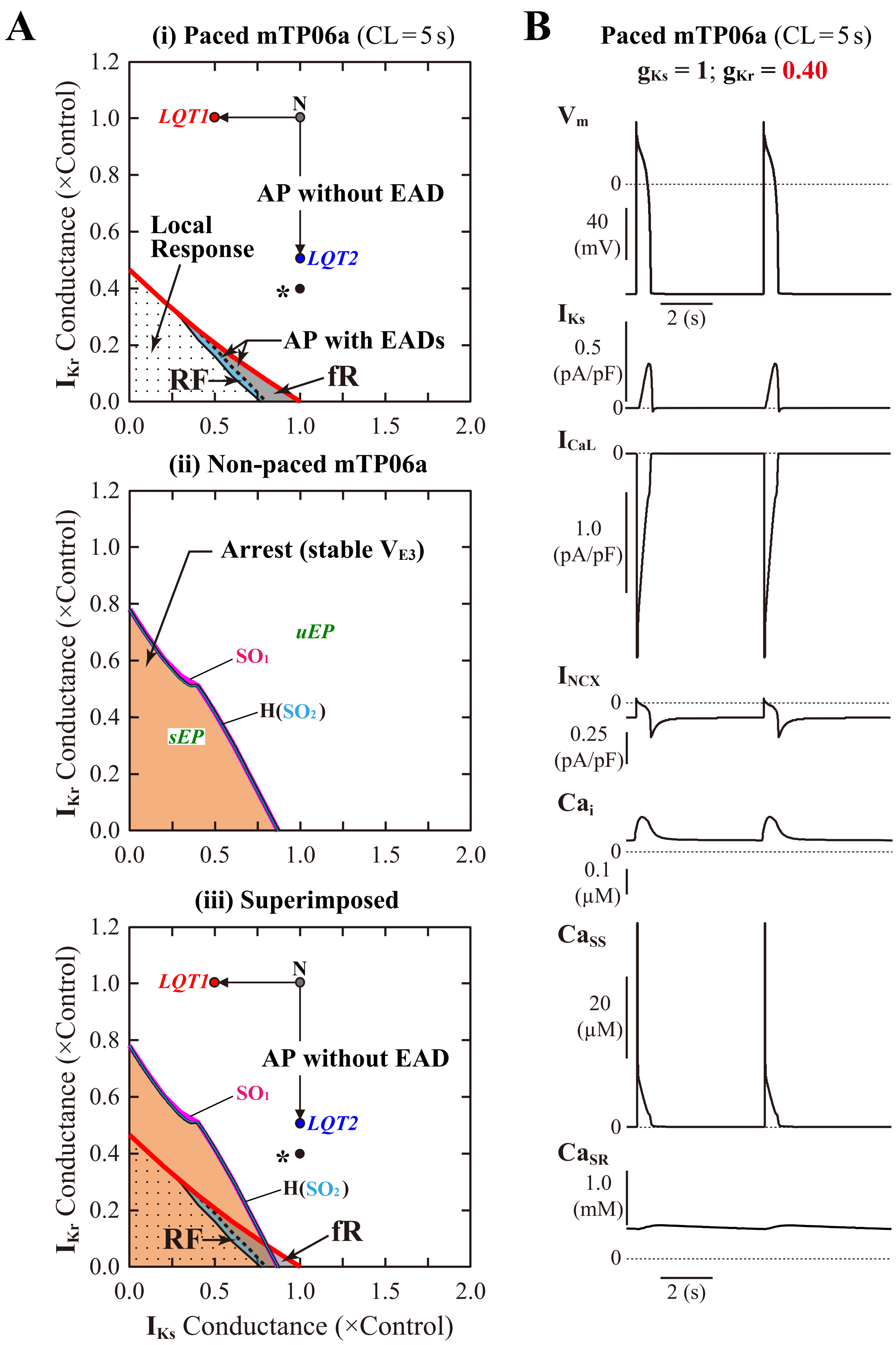

Supplementary Figure S3

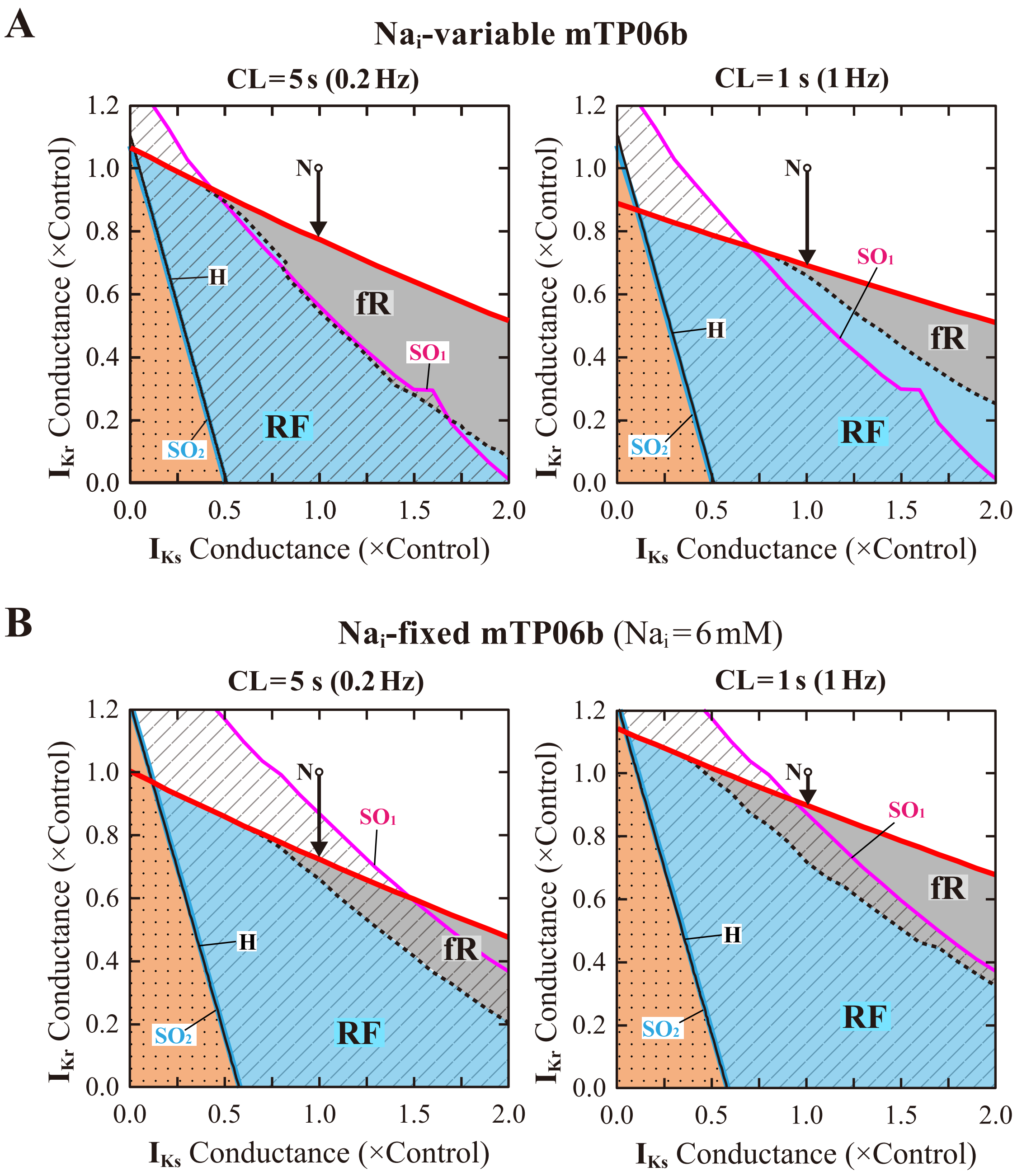

Supplementary Figure S4

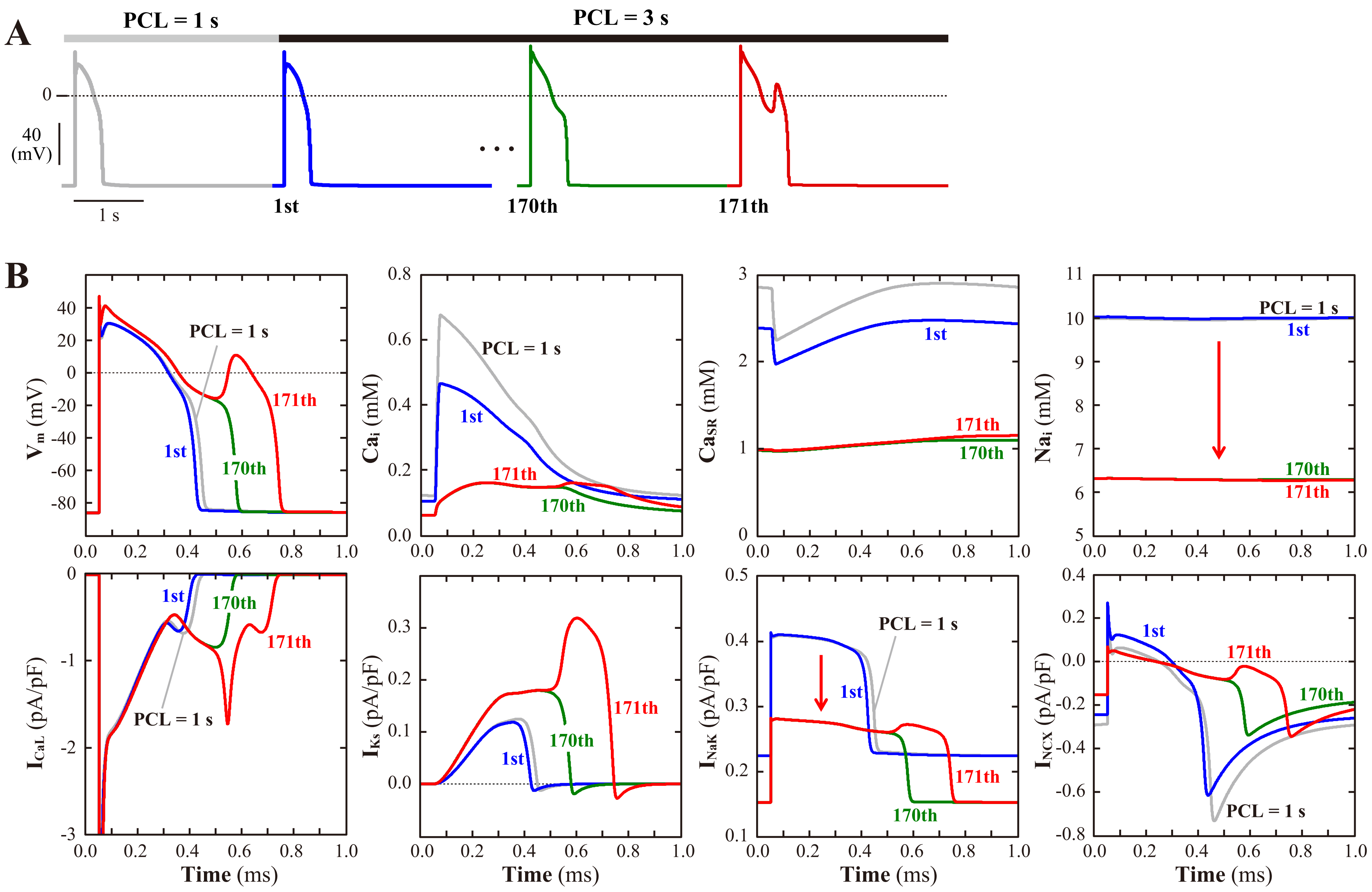

Supplementary Figure S5

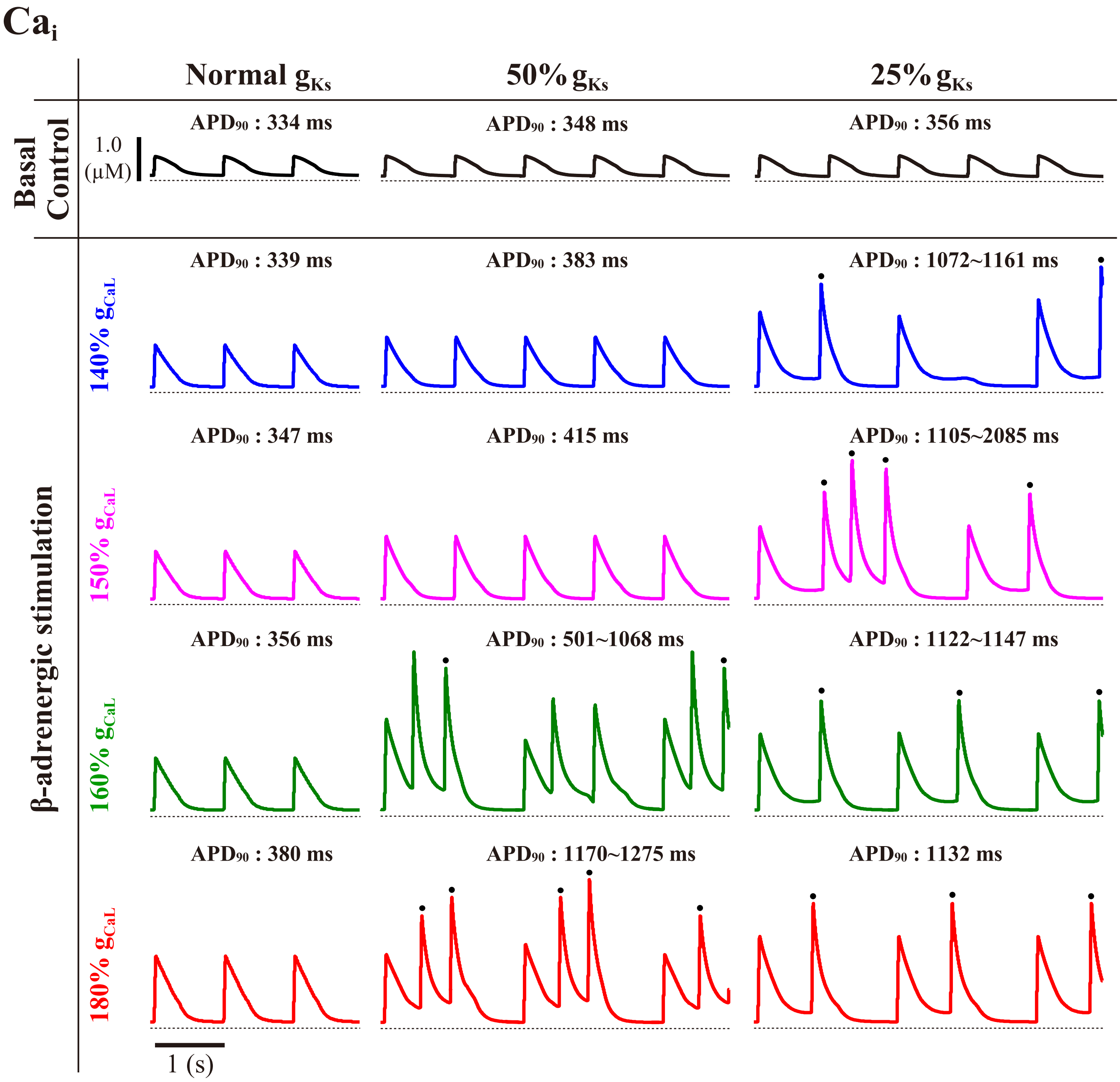

Supplementary Figure S6

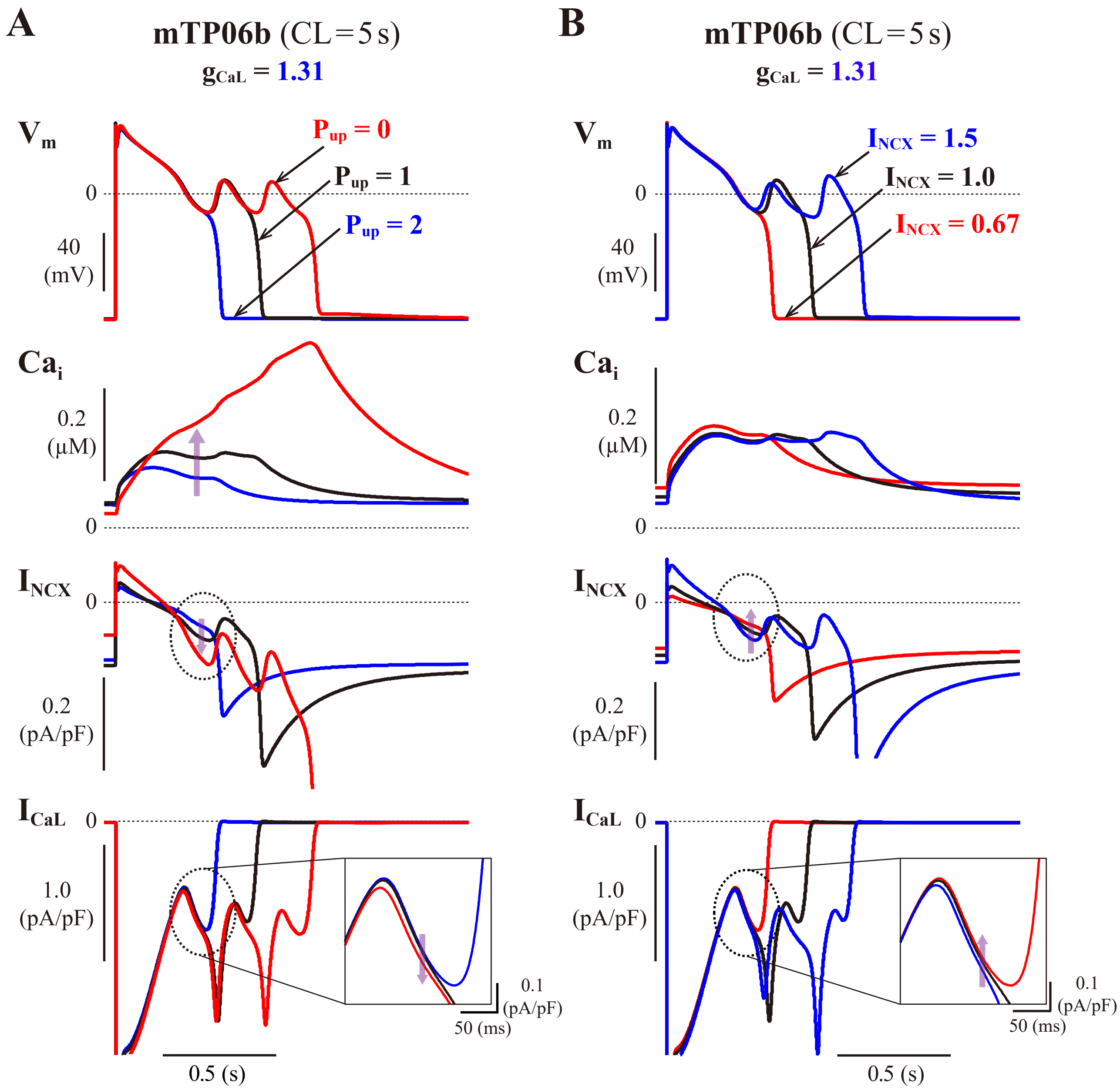

Supplementary Figure S7

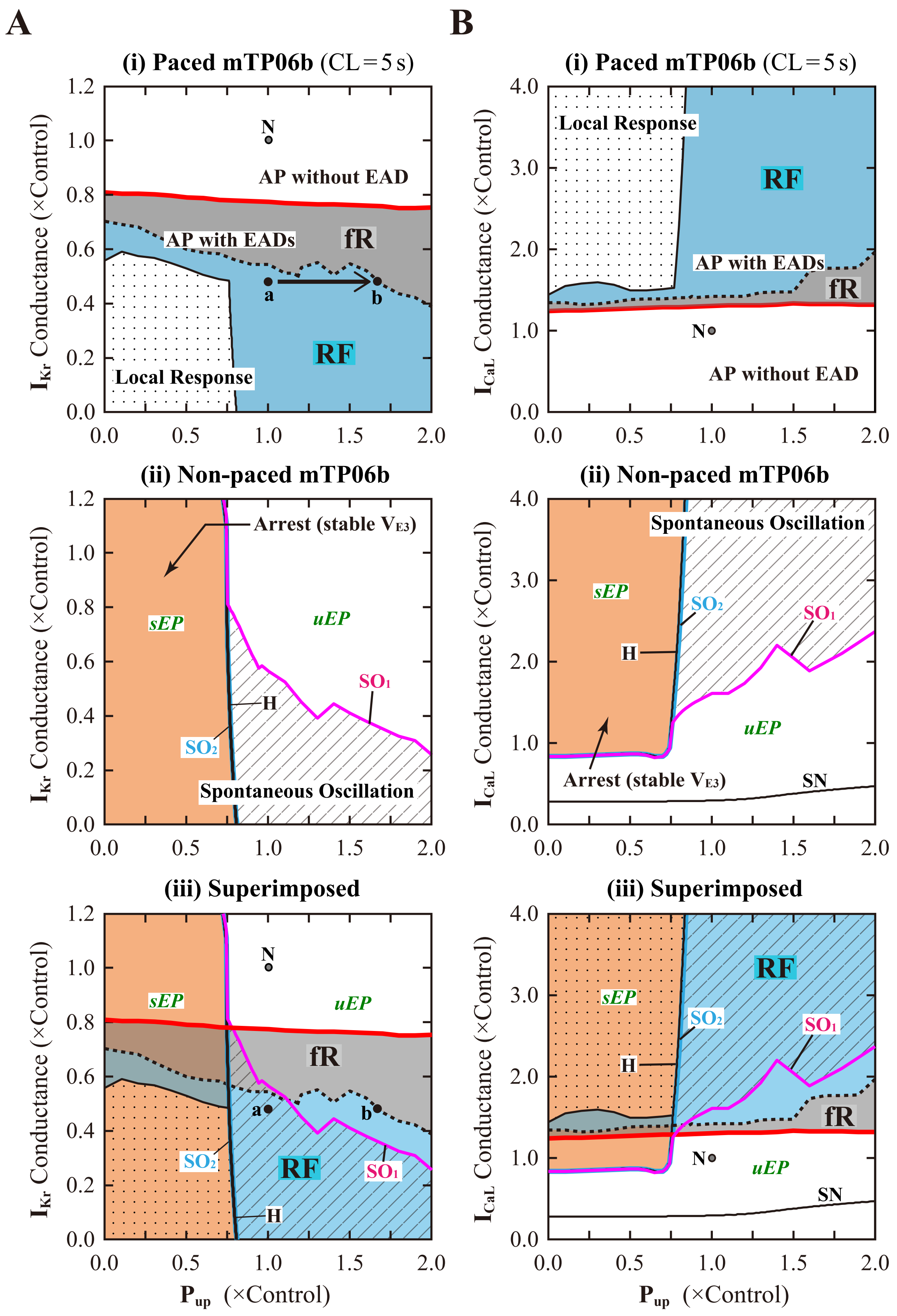

Supplementary Figure S8

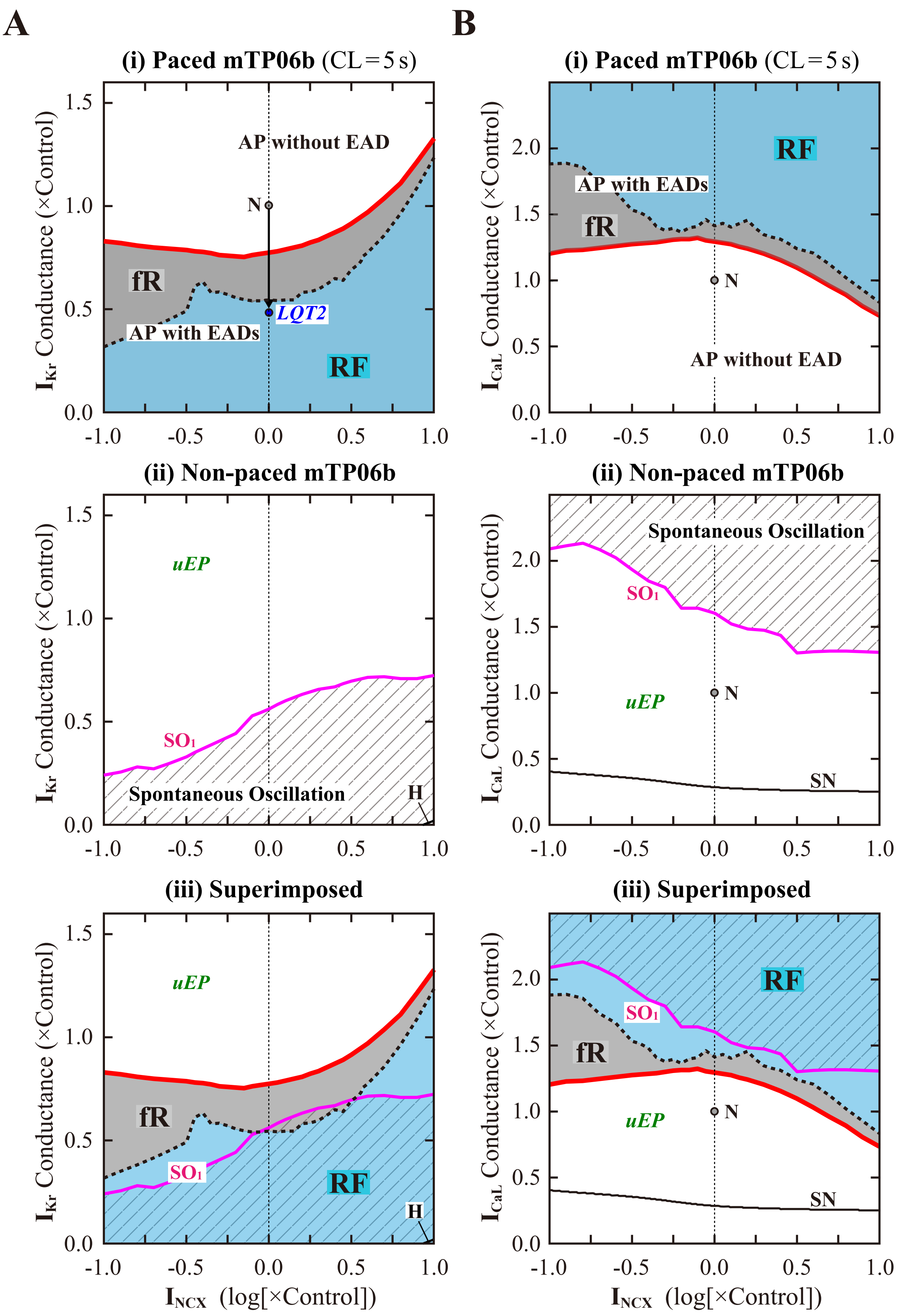

Supplementary Figure S9

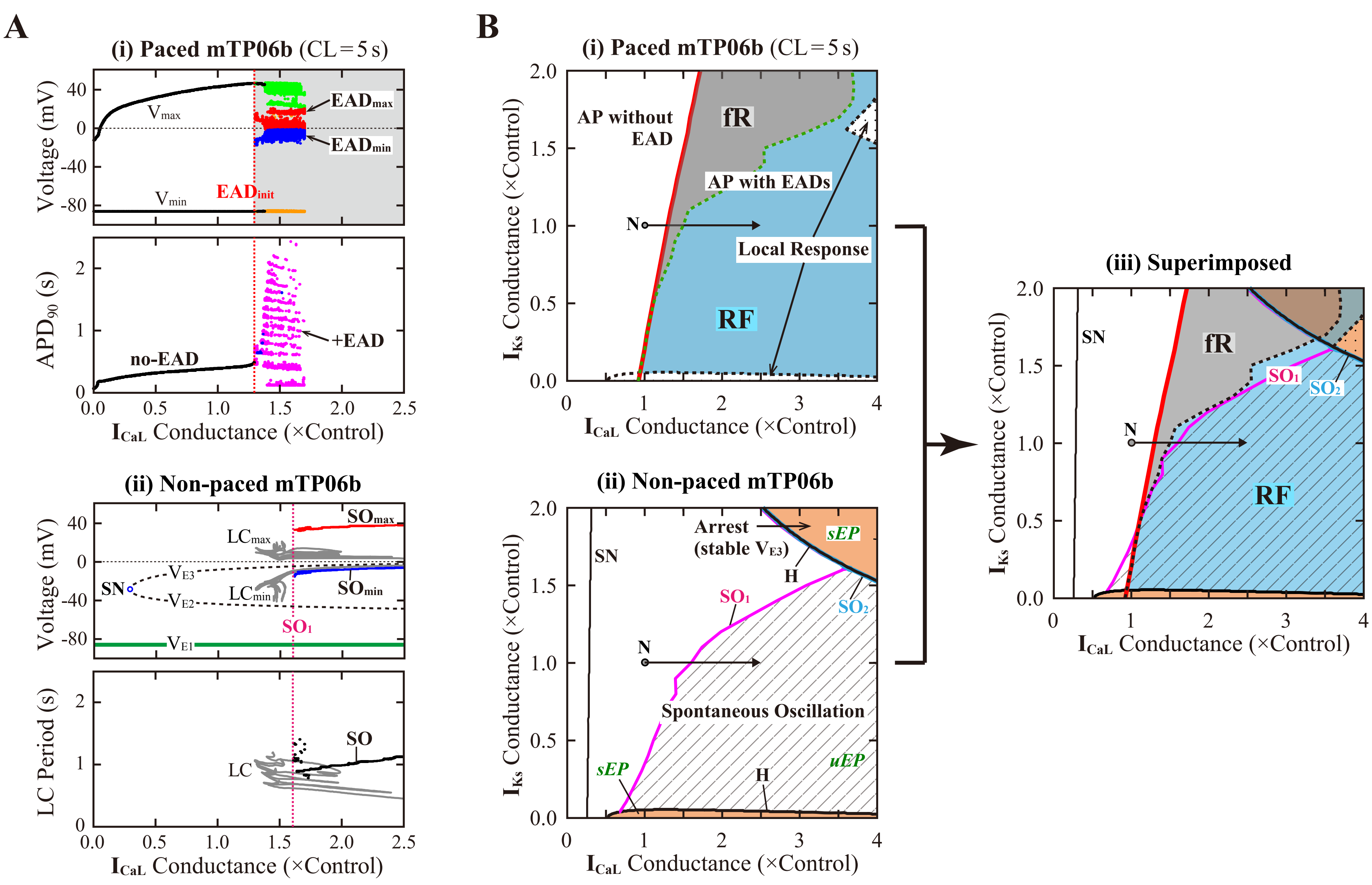

Supplementary Figure S10

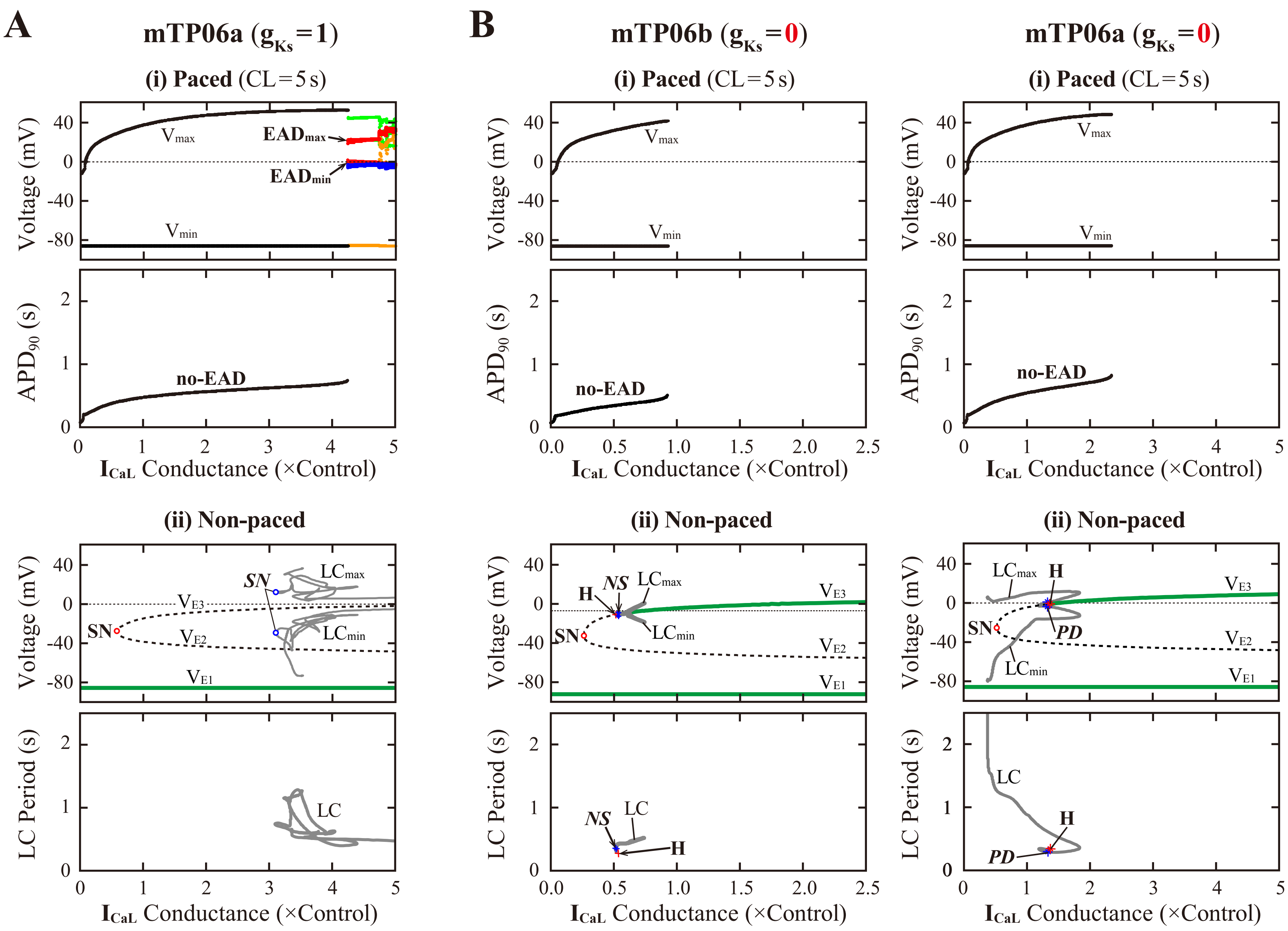

Supplement: Supplementary file 1 [file Data_Sheet_1.pdf]
